# Acute myocardial infarction: Development and application of an ICD-10-CM-based algorithm to a large U.S. healthcare claims-based database

**DOI:** 10.1371/journal.pone.0253580

**Published:** 2021-07-01

**Authors:** Patrick Saunders-Hastings, Sze Wing Heong, Jenny Srichaikul, Hui-Lee Wong, Azadeh Shoaibi, Kinnera Chada, Timothy A. Burrell, Graça M. Dores

**Affiliations:** 1 Gevity Consulting Inc., Ottawa, ON, Canada; 2 Center for Biologics Evaluation and Research, US Food and Drug Administration, Silver Spring, MD, United States of America; 3 IBM Watson Health, Cambridge, MA, United States of America; University of Western Australia, AUSTRALIA

## Abstract

**Background:**

Healthcare administrative claims data hold value for monitoring drug safety and assessing drug effectiveness. The U.S. Food and Drug Administration Biologics Effectiveness and Safety Initiative (BEST) is expanding its analytical capacity by developing claims-based definitions—referred to as *algorithms*—for populations and outcomes of interest. Acute myocardial infarction (AMI) was of interest due to its potential association with select biologics and the lack of an externally validated International Classification of Diseases, 10^th^ Revision, Clinical Modification (ICD-10-CM) algorithm.

**Objective:**

Develop and apply an ICD-10-CM-based algorithm in a U.S. administrative claims database to identify and characterize AMI populations.

**Methods:**

A comprehensive literature review was conducted to identify validated AMI algorithms. Building on prior published methodology and consistent application of ICD-9-CM codes, an ICD-10-CM algorithm was developed via forward-backward mapping using General Equivalence Mappings and refined with clinical input. An AMI population was then identified in the IBM^®^ MarketScan^®^ Research Databases and characterized using descriptive statistics.

**Results and discussion:**

Between 2014–2017, 2.83–3.16 individuals/1,000 enrollees/year received ≥1 AMI diagnosis in any healthcare setting. The 2015 transition to ICD-10-CM did not result in a substantial change in the proportion of patients identified. Average patient age at first AMI diagnosis was 64.9 years, and 61.4% of individuals were male. Unspecified chest pain, hypertension, and coronary atherosclerosis of native coronary vessel/artery were most commonly reported within one day of AMI diagnosis. Electrocardiograms were the most common medical procedure and beta-blockers were the most commonly ordered cardiac medication in the one day before to 14 days following AMI diagnosis. The mean length of inpatient stay was 5.6 days (median 3 days; standard deviation 7.9 days). Findings from this ICD-10-CM-based AMI study were internally consistent with ICD-9-CM-based findings and externally consistent with ICD-9-CM-based studies, suggesting that this algorithm is ready for validation in future studies.

## 1. Introduction

Acute myocardial infarction (AMI) is a common form of coronary artery disease caused by a sudden interruption of blood flow to the heart that results in decreased oxygen supply to the myocardium and clinical or laboratory evidence of myocardial injury. While declines in AMI hospitalization and mortality rates have been observed in recent years [1], it remains an important cause of morbidity and mortality, with an estimated 600,000–750,000 individuals experiencing AMI in the U.S. each year [2,3].

Administrative healthcare claims data hold broad value in enhancing and optimizing clinical health service delivery. In addition to this, public and private health plan administrative claims databases are useful tools for epidemiologic and drug safety studies, giving researchers access to large patient cohorts [4,5]. However, it is important that methods to identify and study patient cohorts be based on the best available evidence to support reliable and valid study findings. Administrative claims classification systems, used in administrative claims data, are useful in defining populations, exposures and outcomes of interest. However, medical product utilization and safety monitoring are not the primary applications of these classification systems, and definitions must be maintained to align with scientific advances in the medical field. To incorporate new and more specific clinical entities, the U.S. ICD system transitioned from the ~14,000-code 9^th^ (ICD-9-CM) to the ~70,000-code 10^th^ (ICD-10-CM) Revision in October 2015, requiring definitions using this coding system standard to be updated. It should be noted that the World Health Organization maintains ICD-10, that other variations of the ICD-10 exist globally and that other countries have adopted ICD-10 at different times.

The U.S. Food and Drug Administration (FDA) Center for Biologics Evaluation and Research (CBER) BEST Initiative is seeking to expand its capacity to assess the safety and effectiveness of biologic products in the U.S. Biologics are preventive and therapeutic products manufactured in living systems, and are distinct from pharmaceuticals (chemically synthesized); biologics include vaccines, blood, allergenics, tissues, and cellular and gene therapies [6]. In support of this effort, CBER has undertaken initiatives to develop new healthcare claims-based definitions (hereafter referred to as “algorithms”) to identify study populations and outcomes of interest. These algorithms should be founded on the best available evidence and incorporate the current healthcare classification systems. AMI is considered a priority outcome due to its clinical importance and associated morbidity and mortality.

Recognizing that the absence of validated ICD-10-CM algorithms can hinder more current administrative claims research in the U.S., we aimed to develop a new healthcare claims-based definition for AMI using ICD-10-CM and to assess the impact of migration from ICD-9-CM to ICD-10-CM diagnosis codes on the identification of AMI in a U.S. administrative claims database. The objectives of this study are therefore 1) to build on prior published AMI algorithms to develop an ICD-10-CM algorithm that identifies AMI in the U.S., 2) to evaluate the feasibility of algorithm use by applying it to a large U.S. administrative healthcare claims database, 3) to characterize the AMI population cohorts to assess clinical plausibility, and to 4) to assess the impact of the U.S. transition to ICD-10-CM on the identification of AMI in a U.S. administrative claims database.

## 2. Methods

### 2.1 Overview

Authors conducted a literature search in April, 2019 (updated May, 2020) to identify relevant claims-based published definitions of AMI, referred to as *algorithms*. Findings informed the development of a draft AMI algorithm that was subsequently refined by consultation with a diverse range of clinical subject matter experts.

### 2.2 Literature search and algorithm development

The aim of this literature search was not to conduct a full systematic review, but to identify the most pertinent literature and best practices to inform algorithm development. Authors first reviewed relevant FDA Sentinel publications available through the public websites (https://www.fda.gov/BiologicsBloodVaccines/ScienceResearch/default.htm; https://www.sentinelinitiative.org/communications/publications). This was followed by a structured review to identify relevant articles in PubMed, Medline and Google Scholar. Pertinent grey literature was identified via searches of publication repositories of relevant organizations, including but not limited to the U.S. Armed Forces Health Surveillance Branch, Agency for Healthcare Research and Quality and Health Canada *Canada Vigilance Program*.

Articles were retained if they reported an administrative claims-based approach for defining AMI (ICD-9 and onwards) with articles from the U.S. and those that reported measures of diagnostic accuracy of most interest. The focus of this coding effort was to identify episodes of AMI so that future studies could employ this algorithm to identify and validate AMI, irrespective of potential underlying cardiovascular risk factors. Therefore, although angina pectoris and chronic ischemic heart disease are closely related conditions and risk factors for AMI, we excluded diagnostic codes related to subsequent episodes of AMI care, intermediate coronary syndrome, old myocardial infarction, angina, and chronic ischemic heart disease.

Findings from the literature review (discussed in **Section 3.1**) informed the development of a draft algorithm. An ICD-10-CM-based algorithm was developed from published applications of ICD-9-CM-based algorithms associated with strong measures of validation performance via forward-backward mapping using General Equivalence Mappings. This process involves translating ICD-9-CM codes to ICD-10-CM (forward-mapping), then translating the identified ICD-10-CM codes back to ICD-9-CM (backward-mapping) to capture codes that may not have one-to-one mapping between versions and to identify additional related codes that may be of relevance. Recent publications have reported that this approach is appropriate for efforts to define AMI using ICD codes [7,8]. The draft algorithm builds on these prior studies, using a more current version of the ICD-10-CM and an approach informed by high-quality ICD-9-CM validation studies, and was subject to further review and refinement by clinical subject matter experts. The clinical review process involved a code-by-code review of all codes included in the algorithm, and of related codes suggested for exclusion, by two independent clinicians (TAB, GMD), who provided feedback on whether each code was likely to be relevant to a true AMI occurrence. Feedback was incorporated in a revised algorithm that was subject to another round of review and approval by these clinicians.

### 2.3 Algorithm characterization study population and analysis

Authors used the final algorithm to execute a series of descriptive analyses to characterize the U.S. AMI population using the IBM MarketScan Research Databases (Commercial and Medicare Supplemental), accessed via the Treatment Pathways platform. The MarketScan Research Databases are a large collection of U.S. healthcare administrative claims data for encounters in emergency, laboratory, pharmacy, inpatient, and outpatient settings that are linked at the individual level. Encounter data include diagnosis, prescription, and procedure information, as well a patient characteristics.

Age- and gender-specific data on enrollment and AMI case counts were extracted from MarketScan Research Databases. Authors restricted analyses to January 1, 2014–December 31, 2017 to span the October 1, 2015 transition from ICD-9-CM to ICD-10-CM. To exclude overt coding errors, ICD-9-CM codes were only queried for January 1, 2014–September 30, 2015, while ICD-10-CM codes were only queried for October 1, 2015–December 31, 2017.

Individuals had to be enrolled continuously in either a commercial or Medicare Supplemental plan to be included in the analysis for a given year. For example, only individuals continuously enrolled from January 1, 2016–December 31, 2016 were included in the “2016” cohort. This was done to avoid errors in proportion calculations associated with including individuals who may have been enrolled at one time but not at the time of the AMI event. Infants (<1 year old) were excluded from proportion calculations as a result of this minimum enrollment requirement.

Analyses were based on counts of individual patients identified through the AMI algorithm, rather than counts of individual AMI events. Thus, individuals were only counted once per query period, using the first event that met the AMI algorithm criteria, regardless of whether multiple AMI events occurred in that individual. As a result, readmissions and subsequent AMI events were excluded from the analysis if they occurred within the same query period (including queries spanning the entire study period). To prioritize algorithm sensitivity and out of concern that restriction of codes based on coding position or healthcare setting could improperly exclude true AMI cases, we considered the presence of a single relevant diagnosis code sufficient for positive AMI identification, irrespective of code position (principal, secondary, unspecified) or healthcare setting (any healthcare setting). Encounters in the following settings were considered in the overall analysis (“any health care setting”): emergency department, inpatient, lab test, non-doctor office visit, other outpatient, other outpatient office visit, primary care physician office visit, pharmacy, and specialty office visit. Analyses of “inpatient setting” AMI and calculation of average length of hospital stay were limited to inpatient encounters. These two patient cohorts (any healthcare setting and inpatient setting) were selected to characterize AMI codes used across the broader healthcare settings (i.e., any healthcare setting) and in the more specific inpatient setting where medications and associated procedures may be of greater interest to health care delivery assessments, depending on the question under study.

Analyses focused on the frequency of AMI diagnoses by calendar year, age and gender, as well as indicators related to concurrent diagnoses, common treatments and length of inpatient hospital stay. We did not consider race and ethnicity, as this information is missing in a substantial proportion of claims. Patient enrollment and counts were summarized using frequencies and proportions for categorical variables and means (and standard deviations [SD]) and medians (and interquartile ranges [IQR]), for continuous variables. Because one of the goals of the study was to provide descriptive statistics of AMI in a U.S. healthcare database, testing for statistical significance was not conducted. Qualitative comparisons were undertaken through visual inspection of quantitative results to assess epidemiologic trends and the impact of the transition to ICD-10-CM.

Concurrent medical conditions were analyzed to assess whether findings were consistent with clinical expectations and between ICD-9-CM and ICD-10-CM cohorts. These conditions were identified based on diagnostic (ICD-9-CM or ICD-10-CM) codes reported within one day of the AMI event. Conditions were categorized by an experienced physician (TAB) into the following categories: cardiac, endocrine, gastrointestinal, hematologic, infectious disease, metabolic, musculoskeletal, neuropsychiatric, pulmonary, renal, respiratory and vascular diseases and factors influencing health status. The same physician also categorized prescription codes into mutually exclusive treatment categories (e.g., cardiac, endocrine, gastrointestinal, hemic, infectious disease, metabolic, neuropsychiatric, nutritional, and pulmonary) according to target organ system. Procedure codes were grouped into evaluation and management (E/M), emergency medical services (EMS), imaging, laboratory, medication/medication management, pathology, procedure, and ultrasound/doppler categories. Concurrent conditions were queried from one day before to one day following AMI diagnosis, while treatments and procedures were queried from one day before to 14 days following AMI diagnosis. These time periods were based on the judgement of clinical subject matter experts (TAB, GMD), and were chosen to limit the inclusion of unrelated diagnoses while capturing potential lags in processing of claims.

Cardiac conditions and drugs identified in the MarketScan Research Databases were of greatest interest. Agents within the cardiac medication category were further classified according to drug class. The frequency of each cardiac drug class was then assessed for individuals receiving an AMI diagnosis in any healthcare setting and individuals receiving an AMI diagnosis in an inpatient setting.

### 2.4 Ethics

The MarketScan Research Databases meet the Health Insurance Portability and Accountability Act requirements for fully de-identified data sets, protecting the privacy of patients and providers. As this study involved the retrospective analysis of de-identified and anonymized data, no ethics approval was required.

## 3. Results

### 3.1 Literature review findings

A total of 16 articles were retained for extraction (see **S1 Appendix**). The use of ICD-9-CM codes 410.x0 (AMI, unspecified episode of care) and 410.x1 (AMI, initial episode of care) to identify potential AMI cases was consistent across studies, and no studies reported improved diagnostic accuracy associated with including procedural or prescription claims. This included a prior U.S. validation study, which was based on a comprehensive review of U.S. and international studies that analyzed administrative claims data used to identify AMI [9–12] and excluded ICD-9-CM 410.x2 (AMI, subsequent episode of care) [13,14]. Cutrona and colleagues [14] limited the search to diagnosis codes in the principal position, and excluded diagnoses for chronic events, ischemic heart disease and prior myocardial infarctions. A positive predictive value (PPV) of 86% (95% confidence interval [CI] 79–91%) was reported, ranging from 76.3–94.3% across data providers. The approach of combining 410.x0 and 410.x1 in the principal diagnosis position has since been applied in other studies [15,16]. A recent chart validation study assessed the accuracy of these codes in a variety of diagnosis positions (based on inpatient hospital encounters) and reported a PPV of 75% overall (95% CI 65–84%), 93% for principal-position diagnoses (95% CI 78–99%), 88% for secondary-position diagnoses (95% CI 72–97%), and 38% for position-unspecified diagnoses (95% CI 20–59%) [17].

One study [18] defined myocardial infarction using a broader range of ICD-9-CM codes, including 410 (AMI), 411 (other acute and subacute forms of ischemic heart disease), 412 (old myocardial infarction), 413 (angina pectoris), 414 (ischemic heart disease), 429.2 (cardiovascular disease, unspecified), and V45.81 (aortocoronary bypass status). The 410 codes were associated with a PPV of 87% (95% CI 60–98%). None of the other codes were associated with a PPV above 25% and were excluded from the current study focused exclusively on AMI.

No ICD-10-CM validation studies specific to the U.S. were found. However, international studies using ICD-10 I21 (acute myocardial infarction) codes were identified [19,20]. In general, ICD-10 algorithms were associated with strong measures of validation performance. For example, a Danish validation study using I21 codes reported a PPV of 97% (95% CI 91–99) [21], while another reported a worst-case and best-case PPV of 74.0% (95% CI 67–81%) and 100% (95% CI 100–100), respectively, associated with principal-position hospital discharge codes [5]. The best-case PPV was calculated by excluding non-retrievable and non-assessable cases, while the worst-case PPV was calculated by including these in the calculation; this was done in order to estimate a PPV range that accounted for the possible sources of error and biases in the non-retrievable and non-assessable cases. Similarly, two Canadian studies using ICD-10 I21 codes suggested that ICD codes were able to correctly identify cases and distinguish between ST-elevation and non-ST elevation AMI events [20,22].

### 3.2 AMI algorithm

The overarching AMI categories selected are summarized in **Table 1**, while the detailed algorithm code list is included in **S2 Appendix**. Any (≥1) of the codes included in **Table 1** were sufficient to warrant a positive case identification, irrespective of healthcare setting or coding position. The algorithm was constructed using only diagnostic coding classification systems (ICD-9-CM and ICD-10-CM). Other classification systems, including procedural (e.g., Healthcare Common Procedure Coding System or Current Procedural Terminology), laboratory (e.g., Logical Observation Identifiers Names and Codes) and prescription (e.g., National Drug Code) classification systems, were not included in the algorithm, in keeping with the approaches described in prior publications [13,23] which found that the addition of other coding classification systems did not improve validation performance and the clinical opinion that AMI was sufficiently recognizable to be captured using diagnosis codes alone.

**Table 1:** ICD-CM codes selected for identification of AMI*.

Code	ICD-CM version and code description
**ICD-9-CM**	
410.x0	AMI, episode of care unspecified
410.x1	AMI, initial episode of care
**ICD-10-CM**	
I21.xx	Acute myocardial infarction
I22.x	Subsequent STEMI and NSTEMI myocardial infarction

*Abbreviations: AMI, acute myocardial infarction; NSTEMI, non-ST elevation myocardial infarction; STEMI, ST elevation myocardial infarction.

### 3.3 Characteristics of AMI cohorts in marketscan research databases

Between 2014–2017 (inclusive), there were 41,172,696 unique individuals in the MarketScan Research Databases (mean age 20.5 years; 48.1% male) continuously enrolled in a health care plan for at least one calendar year, out of 57,305,367 individuals (mean age 30 years; 48.3% male) who were enrolled for at least one day in this period. Overall, 268,424 (0.65%) received at least one AMI diagnosis (ICD-9-CM or ICD-10-CM) in any healthcare setting (**Sections 3.3.1–3.3.4**) and, of these individuals, the majority (63.4%, n = 170,147) received an AMI diagnosis in an inpatient setting. The “inpatient setting” group was examined for concurrent conditions (**Section 3.3.3**), drug orders and procedures (**Section 3.3.4**), and average length of hospital stay (**Section 3.3.5**), with additional analyses for this subpopulation included in **S3 Appendix**.

Demographic characteristics of the enrolled and AMI populations, overall and according to ICD-CM version, are summarized in **Table 2**. Among AMI patients identified in the any healthcare and inpatient settings, individuals 55–64 and 65 years of age or older were disproportionately represented relative to the enrolled population. Seniors (≥65 years) accounted for 44.9% and 42.7% of patients receiving an AMI diagnosis (ICD-9-CM or ICD-10-CM) in any healthcare and inpatient settings, respectively, despite accounting for only 6.7% of all enrolled individuals. The mean age at first AMI diagnosis (ICD-9-CM or ICD-10-CM) was 64.9 years for individuals receiving an AMI diagnosis in any healthcare setting and 65.1 years in the inpatient setting. In addition, there were more AMI cases among males relative to females in both any healthcare setting (61.4% male) and the inpatient setting (61.8% male). Compared to those receiving an ICD-9-CM diagnosis, patients receiving an ICD-10-CM diagnosis in any healthcare setting were slightly older, and a lower proportion were male.

**Table 2:** Summary of demographic profiles for the individuals enrolled in a health care plan for at least one calendar year, those that received at least one AMI diagnosis in any healthcare setting, and those that received an AMI diagnosis in the inpatient setting, overall and according to ICD-9/10-CM version (2014–2017).

Parameter	Enrolled Population	AMI Population (any healthcare setting)			AMI Population (inpatient setting)		
		ICD-9-CM	ICD-10-CM	Combined	ICD-9-CM	ICD-10-CM	Combined
Population Size–N	41,172,696	133,075	156,095	268,424	80,823	95,017	170,147
Mean Age at First Diagnosis–Years (SD)	36.6 (20.5)	64.6 (14.4)	65.4 (14.8)	64.9 (14.7)	64.8 (14.4)	65.6 (14.8)	65.1 (14.6)
Age at First Diagnosis–Years							
0–17 –N (%)	9.048,833 (22.0)	176 (0.1)	233 (0.2)	400 (0.2)	37 (0.1)	62 (0.1)	99 (0.1)
18–34 –N (%)	10,029,259 (24.4)	2,056 (1.6)	2,642 (1.7)	4,587 (1.7)	1,089 (1.4)	1,418 (1.5)	2,495 (1.4)
35–44 –N (%)	6,077,667 (14.8)	6,885 (5.2)	7,553 (4.8)	13,691 (5.1)	4,135 (5.2)	4,491 (4.7)	8,512 (4.8)
45–54 –N (%)	6,748,711 (16.4)	22,094 (16.6)	24,427 (15.7)	43,611 (16.2)	13,676 (16.9)	15,085 (15.9)	28,251 (15.9)
55–64 –N (%)	6,484,285 (15.8)	42,629 (32.0)	49,612 (31.8)	85,699 (31.9)	26,132 (32.3)	30,560 (32.2)	55,257 (31.1)
65+–N (%)	2,783,941 (6.7)	59,235 (44.5)	71,628 (45.9)	120,789 (44.9)	35,754 (44.2)	43,401 (45.7)	75,726 (42.7)
Gender–Male–N (%)	19,800,058 (48.1)	82,805 (62.2)	95,654 (61.3)	164,804 (61.4)	50,389 (62.3)	58,268 (61.3)	105,026 (61.7)

*Abbreviations: AMI, acute myocardial infarction; ICD-CM, International Classification of Diseases, Clinical Modification; N, number. There were 33,216,843 individuals enrolled for at least one calendar year between 2014–2015 (ICD-9-CM era) and 30,319,401 individuals enrolled for at least one calendar year between 2015–2017 (ICD-10-CM era).

#### 3.3.1 Annual frequency and proportion of AMI patients

**Fig 1:**
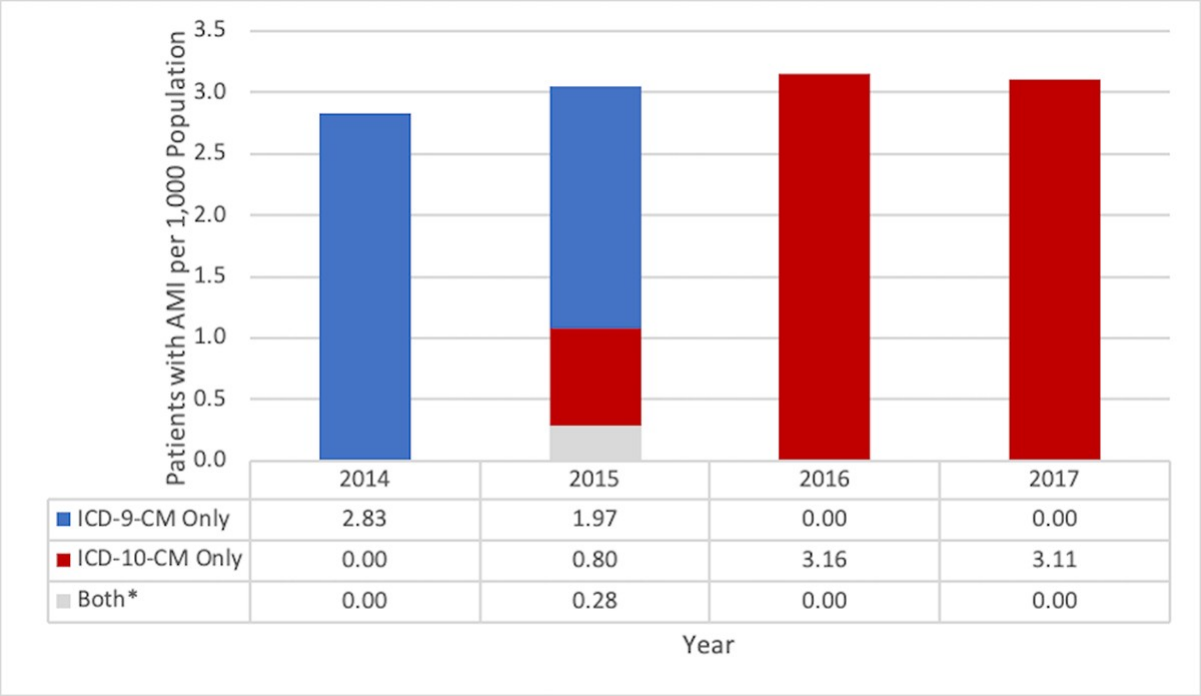
Proportion of patients with AMI per 1,000 enrolled individuals according to year (2014–2017). Abbreviations: AMI, acute myocardial infarction. * Note: In 2015, 6,273 of 67,375 patients (9.3%) received both an ICD-9-CM and ICD-10-CM diagnosis for AMI, in the January–September and October–December timeframe, respectively. These patients were only counted once for proportion estimates (using the “Both” category).

The annual counts of patients with ≥1 diagnosis code for AMI in any healthcare setting during the study period ranged from 61,165 to 80,411, as summarized in **Table 3**. From 2014–2017, 2.83–3.16 individuals/1,000 enrollees/year received ≥1 AMI diagnosis. The annual proportion of enrolled individuals receiving ≥1 AMI diagnosis did not change substantially as a result of the 2015 transition to ICD-10-CM (**Fig 1**). It should be noted that enrollment in the MarketScan Research Databases decreased over time, although, as shown in **Fig 1**, this did not appear to meaningfully impact the proportion of enrolled individuals receiving an AMI diagnosis.

**Table 3:** Annual counts of individuals enrolled in MarketScan Research Databases and counts of patients with ≥1 AMI diagnosis according to ICD-CM codes received in any healthcare setting (2014–2017).

Data Source Code	Calendar Year*			
	2014	2015	2016	2017
	Count (N)	Count (N)	Count (N)	Count (N)
MarketScan Research Databases, enrollment	28,407,959	22,117,235	21,616,367	19,802,253
ICD-9-CM	80,411	49,765		
ICD-10-CM		23,883	68,253	61,165
ICD-9-CM OR ICD-10-CM	80,411	67,375	68,253	61,165

* Blank cells represent that ICD-10-CM codes were not queried in 2014 (prior to the transition to ICD-10-CM) while ICD-9-CM codes were not queried after 2015 (following the transition to ICD-10-CM), as this was excluded from the analysis (refer to Methods, **Section 2.3**).

#### 3.3.2 Age and gender

**Fig 2:**
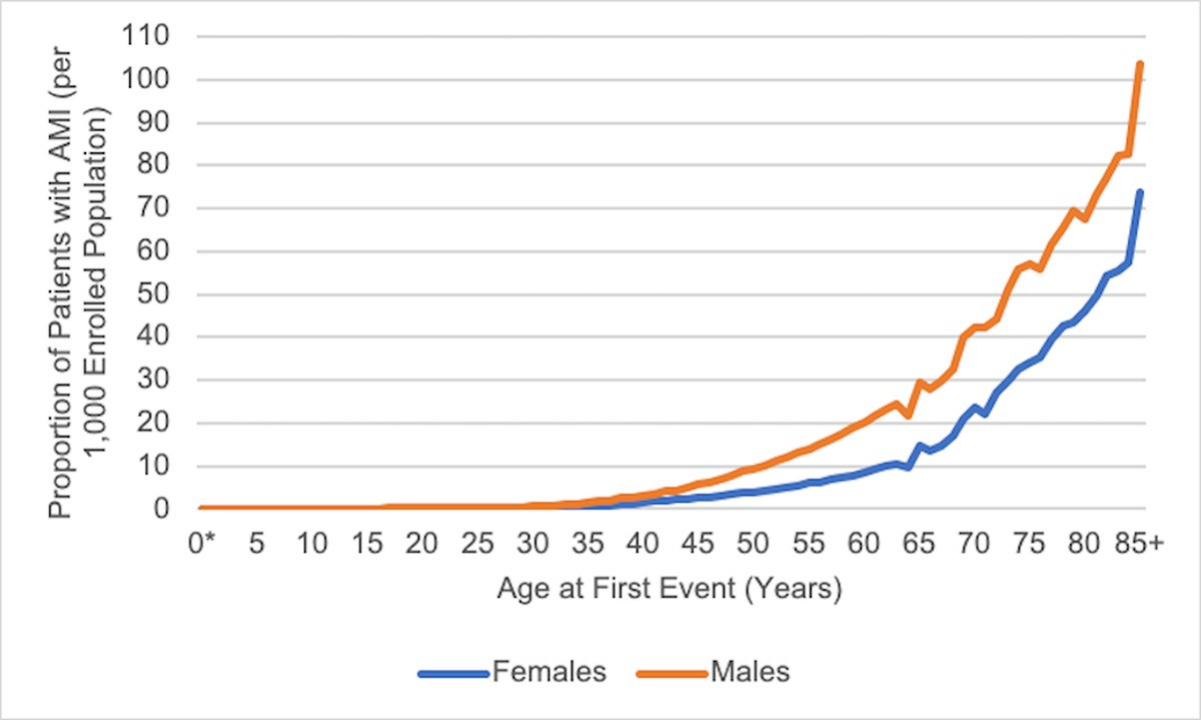
Proportion of patients with at least one diagnosis for AMI in any healthcare setting per 1,000 enrollees, by gender and age at first AMI diagnosis in the database (2014–2017). Abbreviation: AMI, acute myocardial infarction.

The gender-specific proportions of individuals receiving a first AMI diagnosis according to age (**Fig 2**) suggest that the proportion of those experiencing AMI is highest among males, with the number of events increasing beginning at approximately age 40 years among males and females.

#### 3.3.3 Concurrent conditions

The two cohorts of patients receiving ≥1 AMI (either ICD-9-CM or ICD-10-CM) diagnosis codes in any healthcare setting between 2014–2017 were assessed to determine the most frequent diagnosis codes reported in the day immediately before or after receipt of an AMI diagnosis. The ten most common cardiac diagnoses are presented in **Table 4** according to ICD-CM version, while the top 100 ICD-9-CM and ICD-10-CM codes for all disease categories are included in **S3 Appendix (Table C1)**. For both ICD-9-CM and ICD-10-CM, unspecified chest pain (51.3% in ICD-9-CM cohort; 53.6% in ICD-10-CM cohort), essential hypertension (47.0% in ICD-9-CM cohort; 51.6% in ICD-10-CM cohort), and atherosclerosis (46.8% in ICD-9-CM cohort; 43.5% in ICD-10-CM cohort) were the most commonly reported diagnosis codes, in addition to an AMI diagnosis beyond the diagnosis used to identify the individual (i.e., an additional AMI diagnosis code(s) reported within one day of the first).

**Table 4:** Cardiac diagnosis codes most commonly reported the day before or following first AMI diagnosis in any healthcare setting, 2014–2017*.

Code	Diagnosis	Persons	
ICD-9-CM		N	% of Total (N = 133,075)
786.50	Unspecified chest pain	68,311	51.3%
401.9	Unspecified essential hypertension	62,515	47.0%
414.01	Coronary atherosclerosis of native coronary vessel	62,291	46.8%
410.71	Acute myocardial infarction, subendocardial infarction, initial episode of care	58,175	43.7%
410.90	Acute myocardial infarction, unspecified site, episode of care unspecified	47,186	35.5%
272.4	Other and unspecified hyperlipidemia	43,426	32.6%
410.70	Acute myocardial infarction, subendocardial infarction, episode of care unspecified	34,317	25.8%
414.00	Coronary atherosclerosis of unspecified type of vessel, native or graft	32,889	24.7%
794.31	Nonspecific abnormal electrocardiogram [ECG] [EKG]	25,521	19.2%
428.0	Congestive heart failure, unspecified	23,718	17.8%
**ICD-10-CM**		**N**	**% of Total (N = 156,095)**
I21.4	Non-ST elevation (NSTEMI) myocardial infarction	83,648	53.6%
I10	Essential (primary) hypertension	80,495	51.6%
I25.10	Atherosclerotic heart disease native coronary artery w/o angina pectoris	67,948	43.5%
R07.9	Chest pain, unspecified	67,917	43.5%
E78.5	Hyperlipidemia, unspecified	46,708	29/9%
I21.3	ST elevation (STEMI) myocardial infarction of unspecified site	41,291	26.5%
R94.31	Abnormal electrocardiogram [ECG] [EKG]	31,007	19.9%
R07.89	Other chest pain	30,194	19.3%
I50.9	Heart failure, unspecified	21,448	13.7%
I51.7	Cardiomegaly	17,788	11.4%

* Percentages add to more than 100% because a patient could receive more than one diagnosis for a concurrent condition (i.e., diagnosis categories are not mutually exclusive).

Results of the same analyses limited to the inpatient setting (n = 170,147) are detailed in **S3 Appendix (Table C2).** Concurrent cardiac conditions were more common in the inpatient setting, though unspecified chest pain (68.8% in ICD-9-CM cohort; 58.0% in ICD-10-CM cohort), atherosclerosis (62.6% in ICD-9-CM cohort; 56.5% in ICD-10-CM cohort), essential hypertension (56.6% in ICD-9-CM cohort; 62.7% in ICD-10-CM cohort) and additional AMI diagnoses remained most common.

#### 3.3.4 Treatment

The cohorts of patients receiving ≥1 AMI diagnosis codes in any healthcare setting (n = 268,424) and in the inpatient setting (n = 170,147) between 2014–2017 were assessed to examine the most frequent medications and procedures coded one day before through two weeks following an AMI diagnosis. The most commonly reported cardiac medications during this 15-day time period are grouped by drug class and summarized in **Table 5.** The top 100 codes for all treatment categories and the assigned drug class are included in **S3 Appendix (Table C3)** for patients receiving an AMI diagnosis in any healthcare setting. Results of the analysis limited to those receiving the diagnosis in the inpatient setting (n = 170,147) are provided in **S3 Appendix (Table C4).** Receipt of medications from each drug class was more common in the inpatient setting, although the most common drug classes were consistent between individuals receiving an AMI diagnosis in any healthcare setting and in the inpatient setting, with beta-blocker (38.8% in any healthcare setting; 48.6% in inpatient setting), anti-lipids (36.8% in any healthcare setting; 45.3% in inpatient setting) and anti-platelet (29.8% in any healthcare setting; 39.4% in inpatient setting) agents being the most frequently prescribed.

**Table 5:** Most common cardiac medications ordered from one day before to 14 days following first AMI diagnosis according to care settings, 2014–2017*.

Drug Class*	Any Healthcare Setting (N = 268,424)		Inpatient Setting (N = 170,147)	
	Persons in Category (N)	% of Total	Persons in Category (N)	% of Total
Beta-blocker	104,085	38.8%	82,697	48.6%
Anti-lipid	98,800	36.8%	77,077	45.3%
Anti-platelet	79,938	29.8%	66,991	39.4%
ACE/ARB	63,064	23.5%	47,953	28.2%
Anti-anginal	49,365	18.4%	40,734	23.9%
Diuretic	34,373	12.8%	25,629	15.1%
Calcium Channel Blocker	19,784	7.4%	13,353	7.8%
Other	11,537	4.3%	9,020	5.3%

Abbreviations: ACE, Angiotensin-converting-enzyme; ARB, Angiotensin II receptor blockers; N, number.

* Drug classes were calculated using the 100 most commonly reported drug products from one day before to 14 days following the AMI diagnosis, and should not be viewed as a comprehensive assessment of *all* drugs within the drug class received by each patient. Hydrochlorothiazide/lisinopril (classified within the ACE/ARB class) was queried for the “any healthcare setting” analysis but not for the “inpatient setting”, as it did not appear in the list of most commonly reported drugs for this latter cohort.

Percentages add to more than 100% because a patient could receive a prescription for more than one treatment related to more than one drug class.

The most common medication prescribed in any healthcare setting (28.3% of individuals) and the inpatient setting (36.6% of individuals) was atorvastatin calcium. Other drug products of interest included aspirin and nitrates, based on the common clinical practice of these agents being administered for AMI. However, aspirin was only reported in 5.5% of patients in any healthcare setting (7.6% in inpatient cohort), while one nitroglycerin formulation was reported in 15.0% of those in any healthcare setting (20.2% in inpatient cohort).

The most common evaluation and management (E/M) procedures (coded among at least 10% of patients in the 15-day period defined surrounding an AMI diagnosis) are listed in **Table 6**, while the top 100 codes for all procedure categories are included in **S3 Appendix (Table C5).** Results of the analysis limited to patients receiving an AMI diagnosis in an inpatient setting (n = 170,147) are provided in **S3 Appendix (Table C6).** Hospital care and emergency department visits were noted as the most common E/M procedure codes observed (all reported among ≥40% of individuals in both any healthcare setting and the inpatient setting).

**Table 6:** Most common evaluation and management procedures codes assigned in the one day before through 14 days following first AMI diagnosis, any healthcare setting (2014–2017)*.

CPT–Code	CPT–Procedure Description	Persons (N)	% of Total (N-268,424)
99232	Subsequent hospital care, per day, for the evaluation and management of a patient, which requires at least 2 of these 3 key components: An expanded problem focused interval history; An expanded problem focused examination; Medical decision making	143,466	53.4%
99223	Initial hospital care, per day, for the evaluation and management of a patient, which requires these 3 key components: A comprehensive history; A comprehensive examination; and Medical decision making of high complexity. Counseling and/or coordination	139,725	52.0%
99285	Emergency department visit for the evaluation and management of a patient, which requires these 3 key components within the constraints imposed by the urgency of the patient’s clinical condition and/or mental status: A comprehensive history	124,184	46.2%
99233	Subsequent hospital care, per day, for the evaluation and management of a patient, which requires at least 2 of these 3 key components: A detailed interval history; A detailed examination; Medical decision making of high complexity.	113,648	42.3%
99291	Critical care, evaluation and management of the critically ill or critically injured patient; first 30–74 minutes	91,543	34.1%
99214	Office or other outpatient visit for the evaluation and management of an established patient, which requires at least 2 of these 3 key components: A detailed history; A detailed examination; Medical decision making of moderate complexity. Counseling	90,752	33.8%
99239	Hospital discharge day management; more than 30 minutes	77,816	29.0%
99222	Initial hospital care, per day, for the evaluation and management of a patient, which requires these 3 key components: A comprehensive history; A comprehensive examination; and Medical decision making of moderate complexity. Counseling and/or coordination	74,374	27.7%
99238	Hospital discharge day management; 30 minutes or less	66,132	24.6%
99213	Office or other outpatient visit for the evaluation and management of an established patient, which requires at least 2 of these 3 key components: An expanded problem focused history; An expanded problem focused examination	50,580	18.8%
99231	Subsequent hospital care, per day, for the evaluation and management of a patient, which requires at least 2 of these 3 key components: A problem focused interval history; A problem focused examination; Medical decision making that is straightforward	46,970	17.5%

Abbreviation: CPT, Common Procedural Terminology; N, number.

* Note that a patient could receive more than one procedural code, so percentages add up to more than 100%.

Across all procedure categories, three procedure codes for routine electrocardiogram (EKG) were the most common codes observed in any health care setting: interpretation and report only (n = 191,167; 71.1%), tracing only (n = 53,638; 20.0%), and tracing with interpretation and report (n = 44,095; 16.4%). Similar to diagnosis codes, procedure codes are not mutually exclusive and the structure of procedural coding for an EKG in the U.S. allows for various services: one code associated with tracing, interpretation, and reporting (reported for 16.4% of patients), one code associated with the tracing only (reported for 20.0% of patients), and one code associated with interpretation and reporting only (reported for 71.1% of patients). A separate query found that 79.0% (n = 211,930) of patients in any healthcare setting and 89.5% (n = 152,305) of patients in the inpatient setting received at least one of these three codes in the 15-day period of interest.

#### 3.3.5 Mean length of inpatient hospital stay

Of the 170,147 individuals receiving a diagnosis of AMI in the inpatient setting, the mean length of hospital stay was 5.6 days (SD 7.9 days), while the median was 3 days (IQR 2–6 days; range 1–384 days). Overall, 69.9% of patients had a length of stay between 1–5 days, 17.8% had a length of stay between 6–10 days and 6.9% had a length of stay over two weeks.

## 4. Discussion & conclusion

This study aimed to build upon prior AMI studies to develop an algorithm that identifies AMI events using U.S. administrative healthcare claims data. The literature review conducted to inform development of the new AMI algorithm reported strong diagnostic accuracy associated with ICD-9-CM codes 410.x0 (AMI, unspecified episode of care) and 410.x1 (AMI, initial episode of care) but did not identify validation studies involving ICD-10-CM algorithms for AMI. Findings from this literature review were used to inform the development of an ICD-10-CM algorithm which was reviewed by clinical subject matter experts and applied in the MarketScan Research Databases to describe the population receiving AMI diagnosis codes in any healthcare setting and the inpatient setting between 2014 and 2017. Findings related to population demographics, common concurrent diagnoses, treatments, and procedures were generally consistent between ICD versions, with existing literature and with clinical expectations.

While ICD-10-CM AMI algorithms were not identified, a recent systematic review of claims-based definitions of AMI reported that ICD-based algorithms performed well across measures of validation performance [24]. Another systematic review of 30 AMI validation studies reported that over 50% of those using hospital data were associated with a sensitivity ≥86% and PPV ≥93% [25]. Negative predictive value and specificity ranged from 75–99% and 89–99%, respectively. This performance and the consistency with which the ICD-9-CM codes have been used to identify potential AMI cases suggest that the proposed ICD-10-CM algorithm is appropriate to identify and study AMI cases. While several cardiovascular risk algorithms are available [26], we did not pursue risk-based analyses because patient risk factors are often not reliably coded in administrative healthcare claims data. The resulting algorithm incorporated ICD-10-CM codes I21.xx and I22.x in any coding position without requiring additional procedural or prescription codes, and was applied to identify cohorts of patients receiving an AMI diagnosis in any healthcare setting and in the inpatient setting. Not unexpectedly, the majority of individuals (63.4%) received an AMI diagnosis in the inpatient setting.

Between 2014 and 2017, the proportion of individuals receiving an AMI diagnosis did not vary substantially from year to year. Patients receiving an ICD-10-CM diagnosis were slightly older and slightly more likely to be female than those receiving an ICD-9-CM diagnosis, in any healthcare setting and in the inpatient setting. Diagnoses were more common among males and among those over 40 years of age, with an average age at first AMI diagnosis (as reported in the MarketScan Research Databases between 2014 and 2017) of 64.9 years in the any healthcare setting population and 65.1 years in the inpatient setting population. This finding is similar to the overall mean age of 65.2 years reported among patients hospitalized for ST-elevation AMI in California [27], but may have been influenced by the under-representation of seniors (individuals ≥65 years of age) in our database (6.7% of the enrolled population compared to 16% of the 2017 U.S. population) [28]. Another U.S. study of 322,523 patients with an AMI diagnosis in an inpatient setting reported an average age of 61 years, with a higher frequency in men [3,27]. While we did not assess whether AMI symptoms began in the inpatient or outpatient setting, Kaul and colleagues [27] described substantial differences in mean age between AMI with onset in the outpatient (mean age 64.9 years) compared to the inpatient setting (mean age 71.5 years).

We found that the non-AMI diagnosis codes most frequently reported within one day of an AMI diagnosis were chest pain, atherosclerosis and essential hypertension, for both ICD-9-CM and ICD-10-CM analyses and in both any healthcare and inpatient settings. This is consistent with symptoms and known risk factors for AMI and prior studies that report an association between hypertension and an increased risk or attack rate in AMI patients [3,29–32].

Atorvastatin calcium was the most commonly reported drug in the one day before through 14 days following first AMI diagnosis (28.3% of the any healthcare setting cohort; 36.6% of inpatient setting cohort) in our study. While aspirin and nitrates are among the most important medications administered on initial presentation of AMI and other acute coronary syndromes, if not contraindicated, these were not among the most commonly coded medications in our database. In the any healthcare setting, we found one formulation of nitroglycerin and aspirin were reported for 15.0% and 5.5% of AMI patients, respectively. It is possible that the lower than expected reporting of these agents is due to administration prior to arrival in the emergency department, either at home or in an ambulance, or due to these agents not being submitted for reimbursement in administrative claims.

Beta-blockers (metoprolol tartrate, carvedilol, atenolol, and metoprolol succinate) represented the most commonly prescribed drug class for AMI ordered in the 15-day period surrounding AMI diagnosis (38.8% of any healthcare settings cohort; 48.6% of inpatient setting cohort). All drugs among the classes assessed were more frequently ordered among the inpatient setting population than those among the any healthcare setting population. Notably, an antiplatelet agent–clopidogrel–was the second most commonly ordered drug in both the any healthcare setting and inpatient setting populations, with other antiplatelet agents also noted (ticagrelor, prasugrel). Other medication classes commonly indicated in the treatment of AMI included statin therapy and renin-angiotensin-aldosterone system inhibitors, which comprised the majority of remaining medications identified. These findings are consistent with clinical recommendations for treatment of AMI during the period of study [33]. Other studies have included longer timeframes post-hospitalization, with 82% of AMI patients discharged from the hospital between 2001 and 2006 in Germany prescribed a beta-blocker within 90 days of discharge, while 73% were prescribed a statin and 66% were prescribed aspirin [34]. Another study from the Netherlands reported that 86% of patients from a national AMI registry were prescribed a beta-blocker within one year of the AMI event, while 91% were prescribed a statin and 81% were prescribed aspirin [35]. Comparability with our results is limited based on the distinct post-discharge timeframes used across studies as well as differences in data collection methods, clinical practices, and prescription drug coverage.

We also examined common medical procedures coded the day before through 14 days following AMI diagnosis. An EKG is required to diagnose and classify AMI and would be expected in all cases of AMI [36]. An EKG procedure code was identified in 71.1% (interpretation and report only), 20.0% (tracing only) and 16.4% (interpretation and report) of AMI cases. Of the any healthcare setting and inpatient setting populations, 79.0% and 89.5% received at least one of these three codes in the day before and 14 days following an AMI diagnosis, consistent with what is expected in clinical care. Additional EKG procedures may have been missed if performed outside the hospital or if a claim was not submitted by the treating clinician. Given that serial EKGs are typically performed in the AMI patient population, patients may receive multiple EKG procedure codes. We did not identify other studies that assessed the frequency of administrative healthcare claims procedure codes among the general U.S. population that experienced an AMI.

The median length of stay for inpatient admissions was 3 days. This is consistent with data reported by others and in alignment with clinical guidelines indicating that low-risk AMI patients may be safely discharged within 72 hours of admission [19,37,38]. The calculated length of stay is lower than the 4 days observed in acute care hospitals in the Minneapolis–St. Paul metropolitan area in 2001 [37], which may reflect the distinct study periods, given more recent efforts to reduce length of hospital stay associated with AMI [38]. Other reasons for length of stay differences may reflect patient population characteristics (e.g., age), with longer length of stay reported among patients with onset of AMI in the inpatient setting (11.4 days) in contrast to those with onset of AMI in the outpatient setting (4.7 days) between 2008–2011 [27] or geographic differences in practice patterns.

Strengths of this study are the development of an AMI algorithm for ICD-10-CM based on a comprehensive review of AMI coding definitions available in the literature and active engagement with clinical subject matter experts. This effort builds on a prior study by Panozzo and colleagues [8] is informed by high-quality validation studies identified in the literature, uses a more current version of the ICD-10-CM to provide a more comprehensive list of AMI codes, and characterizes AMI events further into the ICD-10-CM era. To assess the plausibility of the algorithm, we applied the algorithm in a large U.S. database utilizing ICD-9-CM and ICD-10-CM to characterize the AMI population and generate descriptive statistics overall and by coding schema. This effort should not be viewed as a substitute for medical chart validation, but may be considered as an initial step toward assessing whether the use of ICD-10-CM codes generated reasonable—and clinically credible—results that are comparable to ICD-9-CM-based algorithms subjected to independent validation.

The study also includes important limitations. First, the AMI algorithm has not been subjected to a medical record-based validation study, and diagnostic accuracy may vary based on healthcare setting and other criteria (such as diagnosis coding position). Although prior AMI studies reported strong validation performance measures for ICD-9-CM algorithms, these may not be directly transferable to the application of this algorithm in the MarketScan Research Databases. In addition, differences in coding standards and practices between ICD-9-CM and ICD-10-CM and across databases, as well as variance in population characteristics and prevalence of AMI, can impact measures of validation performance. As such, the performance characteristics of the algorithm defined herein remain undefined. For the purpose of this study, the algorithm was applied in the most inclusive manner possible, and positive AMI case identification was independent of coding position or healthcare setting (though some analyses focused specifically on diagnoses in the inpatient setting). Thus, more non-AMI cases may have been included than in prior studies that further restricted their study population criteria. We recognize that this more inclusive approach may result in the identification of events associated with particular procedures or conditions, and that future applications of the algorithm may restrict codes based on position or healthcare setting, though this will be dependent on the specific research question. Also, more specific analyses based on care type (e.g., comparing diagnoses in acute, rehabilitative, and palliative settings) or urgency were considered outside the scope of this initial application. Findings in our study may not be representative of the U.S. population, as our analyses were restricted to insured individuals within specified health care plans who may differ in their access to care and health status from the general population. We also were unable to account for the effects of race and ethnicity, as this information was missing in a substantial proportion of claims. Analyses were also based on administrative healthcare claims data, which provide information on medications ordered but do not capture medication indication, dispensation, or consumption, nor do they reflect patient adherence. Lastly, we could not calculate AMI incidence rates because our analyses only considered the first AMI within a given time period, and therefore we cannot exclude the possibility of an individual having an MI prior to entry into the study. The algorithm proposed herein can be applied in other databases to assess incidence rates. However, the characteristics of the AMI population in our study suggest that AMI cases were appropriately identified and that that this algorithm is a candidate for future validation studies utilizing a clinical database.

In conclusion, this study aimed to apply a rigorous approach to develop an algorithm to identify AMI events in administrative claims data. The algorithm developed is based on a comprehensive review of the literature, informed by prior validation studies and refined through a forward-backward crosswalk from ICD-9-CM to ICD-10-CM and substantial clinical consultation. Application of the algorithm in a large administrative claims database supports analyses of population groups that received AMI diagnoses and common concurrent diagnoses, treatments and procedures. Findings are consistent between ICD versions (allowing for the differences in code specificity), with existing literature and with clinician expectations.

## Supporting information

S1 Appendix Literature review summary. (DOCX)

S2 Appendix Acute myocardial infarction algorithm. (DOCX)

S3 Appendix Supplemental analyses. (DOCX)
