# Supplementary material for: Acute myocardial infarction: Development and application of an ICD-10-CM-based algorithm to a large U.S. healthcare claims-based database
Source: PLoS One. 2021 Jul 1;16(7):e0253580. doi: 10.1371/journal.pone.0253580 (PMC8248590; doi:10.1371/journal.pone.0253580)
Supplement: S1 Appendix — (DOCX) [file pone.0253580.s001.docx]

# Appendix A. Literature Review Summary

**Table A1**, below, summarizes results of the 16 studies used to inform the development of the acute myocardial infarction (AMI) algorithm.

**Table A1. Literature review summary**

| **Citation** | **Title** | **Country** | **Summary** | **Disease Definition** | **Algorithm/Criteria** | **Validity** |
| --- | --- | --- | --- | --- | --- | --- |
| Ando et al., 2018 | Positive predictive value of ICD-10 codes for acute myocardial infarction in Japan: a validation study at a single center | Japan | Calculated PPV of acute myocardial infarction diagnoses in Japanese claims. | “definite AMI was defined as the condition satisfying one of the 5 criteria: (i) detection of the rise and/or fall of cardiac biomarkers accompanied by ischemic symptoms, electrocardiographic (ECG) change or imaging evidence; (ii) sudden unexpected cardiac death; (iii) percutaneous coronary intervention (PCI) related MI (iv) coronary artery bypass grafting (CABG) related MI and (v) post-mortem pathologic finding.” (p.3) | ICD-10 I21, I22 or I23 | PPV 82.5% (95% CI 76.5–87.5%) |
| Ammann et al., 2018 | Chart validation of inpatient ICD-9-CM administrative diagnosis codes for acute myocardial infarction (AMI) among intravenous immune globulin (IGIV) users in the Sentinel Distributed Database | USA (Sentinel) | Chart validation study of ICD-9-CM codes for AMI | Third universal definition of myocardial infarction | ICD-9-CM 410.x1 or 410.x0 reported during inpatient hospital encounter. | Inpatient PPV: Overall: 75% (95% CI: 65–84%) Principal position: 93% (95% CI: 78–99%)  Secondary position: 88% (95% CI: 72–97%)  Position unspecified: 38% (95% CI: 20–59%) |
| Austin et al., 2002 | A multicenter study of the coding accuracy of hospital discharge administrative data for patients admitted to cardiac care units in Ontario | Canada | Study used the Canadian Institute of Health Information hospital discharge abstracts to assess the accuracy of coding for AMI | “The Fastrak II CCU discharge diagnosis was considered the gold standard for this study be- cause it had been gathered prospectively at the time of the CCU discharge” (p.291) | ICD-9 410 | PPV 88.5%; Sensitivity 88.8%; Specificity 92.8% |
| Chrischilles et al., 2015 | Mini-Sentinel Assessment Protocol; Thromboembolic events after immunoglobulin administration | USA (Mini-Sentinel) | Protocol for Mini-Sentinel study for validation of thromboembolic events | Third universal definition for myocardial infarction | ICD-9-CM 410.x0, 410.x1 | Not reported |
| Coloma et al., 2013 | Identification of acute myocardial infarction from electronic healthcare records using different disease coding systems: a validation study in three European countries | Denmark, Italy, Netherlands | Calculated PPV of AMI diagnoses | Third universal definition for myocardial infarction | ICD9-CM codes 410/410.x/410.x0 and ICD-10 code I21.x | “ICD-10 codes had a ‘best-case scenario’ PPV of 100% while ICD9-CM codes had a PPV of 96.6% (95% CI 93.2% to 99.9%).” (p.1) |
| Cutrona et al., 2012 | Design for validation of acute myocardial infarction cases in Mini-Sentinel | USA (Mini-Sentinel) | Describe AMI validation study (Cutrona et al., 2013) and outline key decision points. Algorithm used ICD-9-CM, codes 410.x0-410.x1. | Not specified | AMI patients with ICD-9-CM 410.x0 and 410.x1 discharge codes in principal (or first-listed) position. | Not reported (calculated in Cutrona et al., 2013) |
| Cutrona et al., 2013 | Validation of acute myocardial infarction in the Food and Drug Administration’s Mini-Sentinel program | USA (Mini-Sentinel) | Identified potential AMI (ICD-9-CM 410.x0, 410.x1)in 2009 from four Data Partners participating in the Mini-Sentinel Program.   Authors reported PPV of 86.0% (95% CI 79.2–91.2%). PPV ranged from 76.3% to 94.3% across Data Partners. | Criteria from the Joint European Society of Cardiology and American College of Cardiology Global Task Force. | Hospitalized patients with 410.x0 or 410.x1 in primary position | PPV calculated based on definite or probable cases.  Overall, the PPV was 86.0% (95% CI: 79.2–91.2%). PPVs ranged from 76.3% to 94.3% across Data Partners. |
| Davis et al., 2013 | Validation of Diagnostic and Procedural Codes for Identification of Acute Cardiovascular Events in US Veterans with Rheumatoid Arthritis | USA | Study used Veterans Affairs Rheumatoid Arthritis (VARA) registry to assess accuracy of codes for cardiovascular events, including myocardial infarction. | “The case definition of a CV event required documentation by a clinician of a MI” (p.3) | ICD-9-CM codes410.x, 411.x, 412.x, 413.x, 414.x, 429.2, and v45.81 | PPV 87% (95% CI 51–88%); NPV 98%; Sensitivity 94%, Specificity 96% |
| Pajunen et al., 2005 | The validity of the Finnish Hospital Discharge Register and Causes of Death Register data on coronary heart disease | Finland | Assessed the validity of AMI diagnoses relative to the American Heart Association 2003 definition | American Heart Association 2003 definition | ICD-9 code 410 and ICD-10 codes I21–I22 as the main diagnosis or additional diagnosis | PPV 90%; Sensitivity 83% |
| Panozzo et al., 2018 | Early impact of the ICD‐10‐CM transition on selected health outcomes in 13 electronic health care databases in the United States | USA | Mapped ICD-9-CM disease code algorithms to ICD-10-CM using General Equivalence Mappings | NA (no validation) | From literature (I21.x) – Denmark From simple forward mapping using GEMs 2017: ICD-10-CM I21.09, I21.11, I21.19, I21.29, I21.3, I21.4 From simple backward mapping and forward backward mapping using GEMs 2017: ICD-10-CM I21.01, I21.02, I21.09, I21.11, I21.19, I21.21, I21.29, I21.3, I21.4, I22.0, I22.1, I22.2, I22.8, I22.9 | NA (no validation) |
| Patel et al., 2015 | Validity and utility of ICD-10 administrative health data for identifying ST- and non–ST-elevation myocardial infarction based on physician chart review | Canada | Assessed the validity of two ICD-10 definitions acute myocardial infarction (ST-elevation MI and non–ST-elevation MI) | Clinician “interpreted the 12-lead ECG and reviewed peak myocardial enzymes to determine the clinical diagnosis of non-STEMI versus STEMI” (p. E414) | Primary position diagnosis STEMI: ICD-10 codes I21.0–3 NSTEMI: ICD-10 codes I21.4x | Agreement between chart review and algorithm: 92.3% (STEMI) and 100% (NSTEMI) |
| Solomon et al., 2004 | Relationship Between Selective Cyclooxygenase-2 Inhibitors and Acute Myocardial Infarction in Older Adults | USA | Case-control study examining the association between rofecoxib, celecoxib, NSAIDs, and AMI | NA (no validation) | ICD-9-CM 410 in the first or second position | NA (no validation) |
| Sundboll et al., 2016 | Positive predictive value of cardiovascular diagnoses in the Danish National Patient Registry: a validation study | Denmark | Examined the positive predictive value (PPV) of cardiovascular diagnoses in the DNPR | Medical record review was used as the reference standard. | First-time myocardial infarction: ICD10 I21 | PPV 97% (95% CI 91–99%) |
| Toh et al., 2018 | Prospective Postmarketing Surveillance of Acute Myocardial Infarction in New Users of Saxagliptin: A Population-Based Study | USA (Mini-Sentinel) | Conducted sequential assessments to test the association between saxagliptin and AMI | NA (no validation) | Principal hospital discharge diagnosis code 410.x0 or 410.x1 (based on Cutrona et al., 2012, 2013) | NA (no validation) |
| Wahl et al., 2010 | Validation of claims-based diagnostic and procedure codes for cardiovascular and gastrointestinal serious adverse events in a commercially insured population | USA | Study validated AMI administrative claims codes with medical chart review in large commercially insured population | “Experts in cardiology, neurology, and gastroenterology designed validation tools to be applied to patient medical records to capture clinical data relevant to confirmed MI” (p. 598) | Hospitalization with ICD-9 code 410.xx (excluding 410.x2) and either a length of stay of 3–180 days or death. | PPV 88.4% (95% CI 83.2–92.5%) |
| Youngson et al., 2016 | Defining and validating comorbidities and procedures in ICD-10 health data in ST-elevation myocardial infarction patients | Canada | Assessed the accuracy of STEMI diagnosis and comorbidities in Canadian registry of consecutive STEMI patients | Not reported | “ICD-10 codes I21.0∗, I21.1∗, I21.2∗, or I21.3∗ in the most responsible diagnosis field.” (p.2) | “A diagnosis of STEMI was correctly identified in the administrative records for 3043 (94.0%) patients.” (p.1) |
