# Supplementary material for: Acute myocardial infarction: Development and application of an ICD-10-CM-based algorithm to a large U.S. healthcare claims-based database
Source: PLoS One. 2021 Jul 1;16(7):e0253580. doi: 10.1371/journal.pone.0253580 (PMC8248590; doi:10.1371/journal.pone.0253580)
Supplement: S2 Appendix — (DOCX) [file pone.0253580.s002.docx]

# Appendix B. Acute Myocardial Infarction Algorithm

**Table B1**, below, lists the ICD-9-CM and ICD-10-CM codes included in the acute myocardial infarction (AMI) algorithm.

**Table B1. Acute myocardial infarction algorithm**

| **Code** | **Description** | **Code Category** | **Code Type** |
| --- | --- | --- | --- |
| **ICD-9-CM** | | | |
| 410.00 | Acute myocardial infarction of anterolateral wall, episode of care unspecified | DX | 09 |
| 410.01 | Acute myocardial infarction of anterolateral wall, initial episode of care | DX | 09 |
| 410.10 | Acute myocardial infarction of other anterior wall, episode of care unspecified | DX | 09 |
| 410.11 | Acute myocardial infarction of other anterior wall, initial episode of care | DX | 09 |
| 410.20 | Acute myocardial infarction of inferolateral wall, episode of care unspecified | DX | 09 |
| 410.21 | Acute myocardial infarction of inferolateral wall, initial episode of care | DX | 09 |
| 410.30 | Acute myocardial infarction of inferoposterior wall, initial episode of care | DX | 09 |
| 410.31 | Acute myocardial infarction of inferoposterior wall, episode of care unspecified | DX | 09 |
| 410.40 | Acute myocardial infarction of other inferior wall, episode of care unspecified | DX | 09 |
| 410.41 | Acute myocardial infarction of other inferior wall, initial episode of care | DX | 09 |
| 410.50 | Acute myocardial infarction of other lateral wall, episode of care unspecified | DX | 09 |
| 410.51 | Acute myocardial infarction of other lateral wall, initial episode of care | DX | 09 |
| 410.60 | True posterior wall infarction, episode of care unspecified | DX | 09 |
| 410.61 | True posterior wall infarction, initial episode of care | DX | 09 |
| 410.70 | Subendocardial infarction, episode of care unspecified | DX | 09 |
| 410.71 | Subendocardial infarction, initial episode of care | DX | 09 |
| 410.80 | Acute myocardial infarction of other specified sites, episode of care unspecified | DX | 09 |
| 410.81 | Acute myocardial infarction of other specified sites, initial episode of care | DX | 09 |
| 410.90 | Acute myocardial infarction of unspecified site, episode of care unspecified | DX | 09 |
| 410.91 | Acute myocardial infarction of unspecified site, initial episode of care | DX | 09 |
| **ICM-10-CM** | | | |
| I21.01 | ST elevation myocardial infarction (STEMI) involving left main coronary artery | DX | 10 |
| I21.02 | ST elevation myocardial infarction (STEMI) involving left anterior descending coronary artery | DX | 10 |
| I21.09 | ST elevation myocardial infarction (STEMI) involving other coronary artery of anterior wall | DX | 10 |
| I21.11 | ST elevation myocardial infarction (STEMI) involving right coronary artery | DX | 10 |
| I21.19 | ST elevation myocardial infarction (STEMI) involving other coronary artery of inferior wall | DX | 10 |
| I21.21 | ST elevation myocardial infarction (STEMI) involving left circumflex coronary artery | DX | 10 |
| I21.29 | ST elevation myocardial infarction (STEMI) involving other sites | DX | 10 |
| I21.3 | ST elevation myocardial infarction (STEMI) of unspecified site | DX | 10 |
| I21.4 | Non-ST elevation myocardial infarction (NSTEMI) | DX | 10 |
| I21.9 | Acute myocardial infarction, unspecified | DX | 10 |
| I21.A1 | Myocardial infarction type 2 | DX | 10 |
| I21.A9 | Other myocardial infarction type | DX | 10 |
| I22.0 | Subsequent ST elevation myocardial infarction (STEMI) of anterior wall | DX | 10 |
| I22.1 | Subsequent ST elevation myocardial infarction (STEMI) of inferior wall | DX | 10 |
| I22.2 | Subsequent non-ST elevation myocardial infarction (NSTEMI) | DX | 10 |
| I22.8 | Subsequent ST elevation myocardial infarction (STEMI) of other sites | DX | 10 |
| I22.9 | Subsequent ST elevation myocardial infarction (STEMI) of unspecified site | DX | 10 |
