# Supplementary material for: Acute myocardial infarction: Development and application of an ICD-10-CM-based algorithm to a large U.S. healthcare claims-based database
Source: PLoS One. 2021 Jul 1;16(7):e0253580. doi: 10.1371/journal.pone.0253580 (PMC8248590; doi:10.1371/journal.pone.0253580)
Supplement: S3 Appendix — (DOCX) [file pone.0253580.s003.docx]

# Appendix C. Supplemental Analyses

**Table C1** lists the 100 ICD-9-CM and ICD-10-CM diagnoses most commonly reported the day before or after an AMI diagnosis among cohorts of 133,075 and 156,095 patients receiving ≥1 ICD-9-CM or ICD-10-CM AMI diagnosis code, respectively, in any healthcare setting between 2014–2017. Diagnoses are organized alphabetically by class and by the most commonly reported diagnoses within each class.

**Table C1.** Diagnosis codes most commonly reported within a day before or after an AMI diagnosis (any healthcare setting).

| **Code** | **Diagnosis Description** | **Class** | **Persons** | |
| --- | --- | --- | --- | --- |
| **ICD-9-CM** | | | **N** | **% of Total (133,075)** |
| 786.50 | Unspecified chest pain | Cardiac | 68,311 | 51.3% |
| 401.9 | Unspecified essential hypertension | Cardiac | 62,515 | 47.0% |
| 414.01 | Coronary atherosclerosis of native coronary vessel | Cardiac | 62,291 | 46.8% |
| 410.81 | Acute myocardial infarction, subendocardial infarction, initial episode of care | Cardiac | 58,175 | 43.7% |
| 410.90 | Acute myocardial infarction, unspecified site, episode of care unspecified | Cardiac | 47,186 | 35.5% |
| 410.70 | Acute myocardial infarction, subendocardial infarction, episode of care unspecified | Cardiac | 34,317 | 25.8% |
| 414.00 | Coronary atherosclerosis of unspecified type of vessel, native or graft | Cardiac | 32,889 | 24.7% |
| 794.21 | Nonspecific abnormal electrocardiogram [ECG] [EKG] | Cardiac | 25,521 | 19.2% |
| 428.0 | Congestive heart failure, unspecified | Cardiac | 23,718 | 17.8% |
| 410.91 | Acute myocardial infarction, unspecified site, initial episode of care | Cardiac | 21,945 | 16.5% |
| 411.1 | Intermediate coronary syndrome | Cardiac | 20,405 | 15.3% |
| 786.59 | Other chest pain | Cardiac | 19,957 | 15.0% |
| 401.1 | Benign essential hypertension | Cardiac | 16,071 | 12.1% |
| 429.3 | Cardiomegaly | Cardiac | 13,705 | 10.3% |
| 410.41 | Acute myocardial infarction, of other inferior wall, initial episode of care | Cardiac | 10,689 | 8.0% |
| 425.4 | Other primary cardiomyopathies | Cardiac | 9,837 | 7.4% |
| 410.40 | Acute myocardial infarction, of other inferior wall, episode of care unspecified | Cardiac | 9,178 | 6.9% |
| 786.51 | Precordial pain | Cardiac | 8,939 | 6.7% |
| 412 | Old myocardial infarction | Cardiac | 8,377 | 6.3% |
| 458.9 | Hypotension, unspecified | Cardiac | 8,072 | 6.1% |
| 414.8 | Other specified forms of chronic ischemic heart disease | Cardiac | 7,943 | 6.0% |
| 410.11 | Acute myocardial infarction, of other anterior wall, initial episode of care | Cardiac | 7,567 | 5.7% |
| 413.9 | Other and unspecified angina pectoris | Cardiac | 7,512 | 5.6% |
| 410.10 | Acute myocardial infarction, of other anterior wall, episode of care unspecified | Cardiac | 7,139 | 5.4% |
| 414.2 | Chronic total occlusion of coronary artery | Cardiac | 6,675 | 5.0% |
| 780.2 | Syncope and collapse | Cardiac | 6,324 | 4.8% |
| 785.0 | Tachycardia, unspecified | Cardiac | 5,656 | 4.3% |
| 414.9 | Chronic ischemic heart disease, unspecified | Cardiac | 5,317 | 4.0% |
| 410.80 | Acute myocardial infarction, of other specified sites, episode of care unspecified | Cardiac | 5,163 | 3.9% |
| 428.21 | Acute systolic heart failure | Cardiac | 4,699 | 3.5% |
| 429.9 | Heart disease, unspecified | Cardiac | 4,603 | 3.5% |
| 410.00 | Acute myocardial infarction, of anterolateral wall, episode of care unspecified | Cardiac | 4,545 | 3.4% |
| 428.9 | Heart failure, unspecified | Cardiac | 4,305 | 3.2% |
| 416.8 | Other chronic pulmonary heart diseases | Cardiac | 3,973 | 3.0% |
| 401.0 | Malignant essential hypertension | Cardiac | 3,954 | 3.0% |
| 272.4 | Other and unspecified hyperlipidemia | Cardiac - lipid | 43,426 | 32.6% |
| 272.0 | Pure hypercholesterolemia | Cardiac - lipid | 9,247 | 6.9% |
| 427.31 | Atrial fibrillation | Cardiac - rhythm | 19,581 | 14.7% |
| 427.89 | Other specified cardiac dysrhythmias | Cardiac - rhythm | 16,087 | 12.1% |
| 427.1 | Paroxysmal ventricular tachycardia | Cardiac - rhythm | 6,376 | 4.8% |
| 427.9 | Cardiac dysrhythmia, unspecified | Cardiac - rhythm | 5,988 | 4.5% |
| 427.5 | Cardiac arrest | Cardiac - rhythm | 4,224 | 3.2% |
| 424.0 | Mitral valve disorders | Cardiac - valve | 13,363 | 10.0% |
| 424.1 | Aortic valve disorders | Cardiac - valve | 7,961 | 6.0% |
| 424.2 | Tricuspid valve disorders, specified as nonrheumatic | Cardiac - valve | 5,517 | 4.1% |
| 250.00 | Diabetes mellitus without mention of complication, type II or unspecified type, not stated as uncontrolled | Endocrine | 26,895 | 20.2% |
| 244.9 | Unspecified hypothyroidism | Endocrine | 7,708 | 5.8% |
| 250.02 | Diabetes mellitus without mention of complication, type II or unspecified type, uncontrolled | Endocrine | 5,803 | 4.4% |
| V45.82 | Percutaneous transluminal coronary angioplasty status | Factors Influencing Health Status | 9,296 | 7.0% |
| V58.82 | Fitting and adjustment of nonvascular catheter, NEC | Factors Influencing Health Status | 7,766 | 5.8% |
| V45.81 | Aortocoronary bypass status | Factors Influencing Health Status | 7,558 | 5.7% |
| V58.81 | Fitting and adjustment of vascular catheter | Factors Influencing Health Status | 7,262 | 5.5% |
| V15.82 | Personal history of tobacco use, presenting hazards to health | Factors Influencing Health Status | 5,384 | 4.0% |
| V45.89 | Other postprocedural status | Factors Influencing Health Status | 4,256 | 3.2% |
| V58.59 | Long-term (current) use of other medications | Factors Influencing Health Status | 4,225 | 3.2% |
| V17.3 | Family history of ischemic heart disease | Factors Influencing Health Status | 4,061 | 3.1% |
| 530.81 | Esophageal reflux | Gastrointestinal | 9,690 | 7.3% |
| 789.00 | Abdominal pain, unspecified site | Gastrointestinal | 6,372 | 4.8% |
| 787.01 | Nausea with vomiting | Gastrointestinal | 4,172 | 3.1% |
| 285.9 | Anemia, unspecified | Hemic | 10,047 | 7.5% |
| 288.60 | Leukocytosis, unspecified | Hemic | 5,578 | 4.2% |
| 285.1 | Acute post hemorrhagic anemia | Hemic | 4,722 | 3.5% |
| 038.9 | Unspecified septicemia | Infectious disease | 6,345 | 4.8% |
| 599.0 | Urinary tract infection, site not specified | Infectious disease | 6,242 | 4.7% |
| 780.60 | Fever, unspecified | Infectious disease | 4,727 | 3.6% |
| 995.91 | Sepsis | Infectious disease | 4,333 | 3.3% |
| 780.79 | Other malaise and fatigue | Metabolic | 9,360 | 7.0% |
| 278.00 | Obesity, unspecified | Metabolic | 7,468 | 5.6% |
| 790.5 | Other nonspecific abnormal serum enzyme levels | Metabolic - lab | 9,771 | 7.3% |
| 790.6 | Other abnormal blood chemistry | Metabolic - lab | 7,144 | 5.4% |
| 276.8 | Hypopotassemia | Metabolic - lab | 6,087 | 4.6% |
| 276.1 | Hypoosmolality and/or hyponatremia | Metabolic - lab | 4,935 | 3.7% |
| 276.2 | Acidosis | Metabolic - lab | 4,803 | 3.6% |
| 790.99 | Other nonspecific findings on examination of blood | Metabolic - lab | 4,622 | 3.5% |
| 729.5 | Pain in limb | Musculoskeletal | 5,698 | 4.3% |
| 305.1 | Tobacco use disorder | Neuropsychiatric | 13,795 | 10.4% |
| 780.97 | Altered mental status | Neuropsychiatric | 7,061 | 5.3% |
| 300.00 | Anxiety state, unspecified | Neuropsychiatric | 5,083 | 3.8% |
| 780.4 | Dizziness and giddiness | Neuropsychiatric | 5,033 | 3.8% |
| 311 | Depressive disorder, not elsewhere classified | Neuropsychiatric | 4,076 | 3.1% |
| 786.09 | Other dyspnea and respiratory abnormality | Pulmonary | 16,411 | 12.3% |
| 511.9 | Unspecified pleural effusion | Pulmonary | 13,613 | 10.2% |
| 518.81 | Acute respiratory failure | Pulmonary | 13,494 | 10.1% |
| 518.0 | Pulmonary collapse | Pulmonary | 12,594 | 9.5% |
| 793.19 | Other nonspecific abnormal finding of lung field | Pulmonary | 10,984 | 8.3% |
| 496 | Chronic airway obstruction, not elsewhere classified | Pulmonary | 10,726 | 8.1% |
| 486 | Pneumonia, organism unspecified | Pulmonary | 10,724 | 8.1% |
| 514 | Pulmonary congestion and hypostasis | Pulmonary | 8,365 | 6.3% |
| 799.02 | Hypoxemia | Pulmonary | 6,352 | 4.8% |
| 518.89 | Other diseases of lung, not elsewhere classified | Pulmonary | 5,591 | 4.2% |
| 786,2 | Cough | Pulmonary | 5,441 | 4.1% |
| 327.23 | Obstructive sleep apnea (adult) (pediatric) | Pulmonary | 4,853 | 3.6% |
| 584.9 | Acute kidney failure, unspecified | Renal | 15,512 | 11.7% |
| 585.9 | Chronic kidney disease, unspecified | Renal | 5,354 | 4.0% |
| 585.3 | Chronic kidney disease, Stage III (moderate) | Renal | 4,987 | 3.7% |
| 403.90 | Hypertensive chronic kidney disease, unspecified, with chronic kidney disease stage I through stage IV, or unspecified | Renal | 4,970 | 3.7% |
| 786.05 | Shortness of breath | Respiratory | 30,506 | 22.9% |
| 443.9 | Peripheral vascular disease, unspecified | Vascular | 4,243 | 3.2% |
| 434.91 | Unspecified cerebral artery occlusion with cerebral infarction | Vascular | 4,162 | 3.1% |
| 433.10 | Occlusion and stenosis of carotid artery without mention of cerebral infarction | Vascular | 4,024 | 3.0% |
| **ICD-10-CM** | | | **N** | **% of Total (N=156,095)** |
| I21.4 | Non-ST elevation (NSTEMI) myocardial infarction | Cardiac | 83,648 | 53.6% |
| I25.10 | Atherosclerotic heart disease native coronary artery w/o angina pectoris | Cardiac | 67,948 | 43.5% |
| R07.9 | Chest pain, unspecified | Cardiac | 67,917 | 43.5% |
| I21.3 | ST elevation (STEMI) myocardial infarction of unspecified site | Cardiac | 41,291 | 26.5% |
| R94.31 | Abnormal electrocardiogram [ECG] [EKG] | Cardiac | 31,007 | 19.9% |
| R07.89 | Other chest pain | Cardiac | 30,194 | 19.3% |
| I50.9 | Heart failure, unspecified | Cardiac | 21,448 | 13.7% |
| I51.7 | Cardiomegaly | Cardiac | 17,788 | 11.4% |
| I21.19 | ST elevation myocardial infarction involving other coronary artery inferior wall | Cardiac | 16,144 | 10.3% |
| I25.110 | ASHD of native coronary artery with unstable angina pectoris | Cardiac | 14,462 | 9.3% |
| I21.09 | ST elevation myocardial infarction involving other coronary artery of ant wall | Cardiac | 12,966 | 8.3% |
| I20.0 | Unstable angina | Cardiac | 10,226 | 6.6% |
| I95.9 | Hypotension, unspecified | Cardiac | 9,564 | 6.1% |
| R07.2 | Precordial pain | Cardiac | 8,781 | 5.6% |
| I25.5 | Ischemic cardiomyopathy | Cardiac | 8,732 | 5.6% |
| I24.9 | Acute ischemic heart disease, unspecified | Cardiac | 8,528 | 5.5% |
| I25.2 | Old myocardial infarction | Cardiac | 8,374 | 5.4% |
| I42.9 | Cardiomyopathy, unspecified | Cardiac | 7,667 | 4.9% |
| R55 | Syncope and collapse | Cardiac | 7,610 | 4.9% |
| R50.21 | Acute systolic (congestive) heart failure | Cardiac | 7,415 | 4.8% |
| I25.119 | ASHD of native coronary artery with unspecified angina pectoris | Cardiac | 7,389 | 4.7% |
| I21.29 | ST elevation (STEMI) myocardial infarction involving other sites | Cardiac | 7,154 | 4.6% |
| I21.11 | ST elevation (STEMI) myocardial infarct involving right coronary artery | Cardiac | 5,911 | 3.8% |
| I21.02 | ST elevation myocardial infarction involving left ant descending coronary art | Cardiac | 5,712 | 3.7% |
| I20.9 | Angina pectoris, unspecified | Cardiac | 5,403 | 3.5% |
| I50.23 | Acute on chronic systolic (congestive) heart failure | Cardiac | 5,294 | 3.4% |
| I25.82 | Chronic total occlusion of coronary artery | Cardiac | 5,050 | 3.2% |
| R09.89 | Other specified symptoms and signs involving circulatory and resp systems | Cardiac - and pulmonary | 6,412 | 4.1% |
| I10 | Essential (primary) hypertension | Cardiac - HTN | 80,495 | 51.6% |
| I11.0 | Hypertensive heart disease with heart failure | Cardiac - HTN | 5,940 | 3.8% |
| E78.5 | Hyperlipidemia, unspecified | Cardiac - lipid | 46,708 | 29.9% |
| E78.0 | Pure hypercholesterolemia | Cardiac - lipid | 8,320 | 5.3% |
| E78.2 | Mixed hyperlipidemia | Cardiac - lipid | 6,628 | 4.2% |
| I48.91 | Unspecified atrial fibrillation | Cardiac - rhythm | 17,745 | 11.4% |
| R00.0 | Tachycardia, unspecified | Cardiac - rhythm | 11,407 | 7.3% |
| I48.0 | Paroxysmal atrial fibrillation | Cardiac - rhythm | 11,097 | 7.1% |
| R00.1 | Bradycardia, unspecified | Cardiac - rhythm | 9,331 | 6.0% |
| I47.2 | Ventricular tachycardia | Cardiac - rhythm | 6,917 | 4.4% |
| I49.9 | Cardiac arrhythmia, unspecified | Cardiac - rhythm | 6,597 | 4.2% |
| I46.9 | Cardiac arrest, cause unspecified | Cardiac - rhythm | 5,483 | 3.5% |
| I34.0 | Nonrheumatic mitral (valve) insufficiency | Cardiac - valve | 12,546 | 8.0% |
| I36.1 | Nonrheumatic tricuspid (valve) insufficiency | Cardiac - valve | 6,205 | 4.0% |
| I35.0 | Nonrheumatic aortic (valve) stenosis | Cardiac - valve | 5,231 | 3.4% |
| E11.9 | Type 2 diabetes mellitus without complications | Endocrine | 27,512 | 17.6% |
| E03.9 | Hypothyroidism, unspecified | Endocrine | 9,559 | 6.1% |
| E11.65 | Type 2 diabetes mellitus with hyperglycemia | Endocrine | 9,132 | 5.9% |
| Z46.82 | Encounter for fitting and adjustment of non-vascular catheter | Factors influencing health status | 10,308 | 6.6% |
| Z45.2 | Encounter for adjustment and management of vascular access device | Factors influencing health status | 9,022 | 5.8% |
| Z87.891 | Personal history of nicotine dependence | Factors influencing health status | 8,300 | 5.3% |
| Z95.1 | Presence of aortocoronary bypass graft | Factors influencing health status | 7,857 | 5.0% |
| Z95.5 | Presence of coronary angioplasty implant and graft | Factors influencing health status | 6,607 | 4.2% |
| Z79.899 | Other long-term drug therapy | Factors influencing health status | 6,531 | 4.2% |
| Z79.82 | Long term (current) use of aspirin | Factors influencing health status | 6,243 | 4.0% |
| Z74.3 | Need for continuous supervision | Factors influencing health status | 5,777 | 3.7% |
| Z82.49 | Family Hx of ischemic heart disease & other diseases of circulatory system | Factors influencing health status | 5,713 | 3.7% |
| Z01.818 | Encounter for other preprocedural examination | Factors influencing health status | 5,233 | 3.4% |
| Z98.89 | Other specified postprocedural state | Factors influencing health status | 5,076 | 3.3% |
| Z21.9 | Gastro-esophageal reflux disease without esophagitis | Gastrointestinal | 11,840 | 7.6% |
| R10.9 | Unspecified abdominal pain | Gastrointestinal | 6,799 | 4.4% |
| D64.9 | Anemia, unspecified | Hemic | 12,082 | 7.7% |
| D78.29 | Elevated white blood cell count, unspecified | Hemic | 7,923 | 5.1% |
| D62 | Acute post hemorrhagic anemia | Hemic | 6,311 | 4.0% |
| A41.9 | Sepsis, unspecified organism | Infectious disease | 10,767 | 6.9% |
| N39.0 | Urinary tract infection, site not specified | Infectious disease | 7,095 | 4.5% |
| R50.9 | Fever, unspecified | Infectious disease | 5,520 | 3.5% |
| R53.1 | Weakness | Metabolic | 9,874 | 6.3% |
| E66.9 | Obesity, unspecified | Metabolic | 9,286 | 5.9% |
| E66.01 | Morbid (severe) obesity due to excess calories | Metabolic | 5,395 | 3.5% |
| R79.89 | Other specified abnormal finding of blood chemistry | Metabolic - laboratory | 16,169 | 10.4% |
| R74.8 | Abnormal levels of other serum enzymes | Metabolic - laboratory | 9,902 | 6.3% |
| E87.2 | Acidosis | Metabolic - laboratory | 8,862 | 5.7% |
| E87.6 | Hypokalemia | Metabolic - laboratory | 8,219 | 5.3% |
| E87.1 | Hypo-osmolality and hyponatremia | Metabolic - laboratory | 6,364 | 4.1% |
| F17.210 | Nicotine dependence, cigarettes, uncomplicated | Neuropsychiatric | 9,919 | 6.4% |
| R41.82 | Altered mental status, unspecified | Neuropsychiatric | 8,713 | 5.6% |
| F41.9 | Anxiety disorder, unspecified | Neuropsychiatric | 6,260 | 4.0% |
| R42 | Dizziness and giddiness | Neuropsychiatric | 5,682 | 3.6% |
| F32.9 | Major depressive disorder, single episode, unspecified | Neuropsychiatric | 5,043 | 3.2% |
| I63.9 | Cerebral infarction, unspecified | Neuropsychiatric | 4,991 | 3.2% |
| R06.02 | Shortness of breath | Pulmonary | 35,869 | 23.0% |
| R91.8 | Other nonspecific abnormal finding of lung field | Pulmonary | 18,047 | 11.6% |
| J90 | Pleural effusion, not elsewhere classified | Pulmonary | 17,404 | 11.1% |
| J98.11 | Atelectasis | Pulmonary | 15,770 | 10.1% |
| R06.00 | Dyspnea, unspecified | Pulmonary | 14,407 | 9.2% |
| J18.9 | Pneumonia, unspecified organism | Pulmonary | 12,985 | 8.3% |
| J96.01 | Acute respiratory failure with hypoxia | Pulmonary | 12,719 | 8.1% |
| J44.9 | Chronic obstructive pulmonary disease, unspecified | Pulmonary | 11,786 | 7.6% |
| J96.00 | Acute respiratory failure, unspecified whether hypoxia or hypercapnia | Pulmonary | 8,758 | 5.6% |
| J81.1 | Chronic pulmonary edema | Pulmonary | 8,210 | 5.3% |
| R09.02 | Hypoxemia | Pulmonary | 7,641 | 4.9% |
| R05 | Cough | Pulmonary | 6,884 | 4.4% |
| G47.33 | Obstructive sleep apnea (adult) (pediatric) | Pulmonary | 6,758 | 4.3% |
| J96.90 | Respiratory failure, unspecified, unspecified whether hypoxia or hypercapnia | Pulmonary | 6,428 | 4.1% |
| J98.4 | Other disorders of lung | Pulmonary | 5,202 | 3.3% |
| J44.1 | Chronic obstructive pulmonary disease with (acute) exacerbation | Pulmonary | 5,068 | 3.2% |
| N17.9 | Acute kidney failure, unspecified | Renal | 20,683 | 13.3% |
| I12.9 | Hypertensive chronic kidney disease stage 1 through stage 4 or unspecified | Renal | 7,423 | 4.8% |
| N18.3 | Chronic kidney disease, stage 3 (moderate) | Renal | 6,987 | 4.5% |
| N18.9 | Chronic kidney disease, unspecified | Renal | 5,879 | 3.8% |
| E86.0 | Dehydration | Renal | 5,414 | 3.5% |

**Table C2** lists the 100 ICD-9-CM and ICD-10-CM diagnoses most commonly reported in the day before or after an AMI diagnosis among cohorts of 80,823 and 95,017 patients receiving ≥1 ICD-9-CM or ICD-10-CM AMI diagnosis code, respectively, in an inpatient setting between 2014–2017. Diagnoses are organized alphabetically by class, then by the most commonly reported diagnoses within each class.

**Table C2.** Diagnosis codes most commonly reported within a day before or after first AMI diagnosis (inpatient setting).

| **Code** | **Diagnosis Description** | **Class** | **Persons** | |
| --- | --- | --- | --- | --- |
| **ICD-9-CM** | | | **N** | **% of Total (80,823)** |
| 786.50 | Unspecified chest pain | Cardiac | 55,576 | 68.8% |
| 410.71 | Acute myocardial infarction, subendocardial infarction, initial episode of care | Cardiac | 50,843 | 62.9% |
| 414.01 | Coronary atherosclerosis of native coronary vessel | Cardiac | 50,634 | 62.6% |
| 401.9 | Unspecified essential hypertension | Cardiac | 45,706 | 56.6% |
| 410.70 | Acute myocardial infarction, subendocardial infarction, episode of care unspecified | Cardiac | 27,012 | 33.4% |
| 410.90 | Acute myocardial infarction, unspecified site, episode of care unspecified | Cardiac | 25,519 | 31.6% |
| 414.00 | Coronary atherosclerosis of unspecified type of vessel, native or graft | Cardiac | 22,340 | 27.6% |
| 428.0 | Congestive heart failure, unspecified | Cardiac | 19,431 | 24.0% |
| 794.31 | Nonspecific abnormal electrocardiogram [ECG] [EKG] | Cardiac | 19,139 | 23.7% |
| 410.91 | Acute myocardial infarction, unspecified site, initial episode of care | Cardiac | 18,208 | 22.5% |
| 411.1 | Intermediate coronary syndrome | Cardiac | 17,466 | 21.6% |
| 786.59 | Other chest pain | Cardiac | 15,232 | 18.8% |
| 401.1 | Benign essential hypertension | Cardiac | 11,409 | 14.1% |
| 429.3 | Cardiomegaly | Cardiac | 11,341 | 14.0% |
| 410.41 | Acute myocardial infarction, of other inferior wall, initial episode of care | Cardiac | 9,616 | 11.9% |
| 425.4 | Other primary cardiomyopathies | Cardiac | 7,860 | 9.7% |
| 786.51 | Precordial pain | Cardiac | 7,269 | 9.0% |
| 458.9 | Hypotension, unspecified | Cardiac | 7,166 | 8.9% |
| 410.11 | Acute myocardial infarction, of other anterior wall, initial episode of care | Cardiac | 6,653 | 8.2% |
| 414.2 | Chronic total occlusion of coronary artery | Cardiac | 6,322 | 7.8% |
| 414.8 | Other specified forms of chronic ischemic heart disease | Cardiac | 6,295 | 7.8% |
| 412 | Old myocardial infarction | Cardiac | 5,964 | 7.4% |
| 413.9 | Other and unspecified angina pectoris | Cardiac | 5,582 | 6.9% |
| 410.40 | Acute myocardial infarction, of other inferior wall, episode of care unspecified | Cardiac | 4,746 | 5.9% |
| 785.0 | Tachycardia, unspecified | Cardiac | 4,665 | 5.8% |
| 780.2 | Syncope and collapse | Cardiac | 4,630 | 5.7% |
| 428.21 | Acute systolic heart failure | Cardiac | 4,391 | 5.4% |
| 414.9 | Chronic ischemic heart disease, unspecified | Cardiac | 4,082 | 5.1% |
| 410.10 | Acute myocardial infarction, of other anterior wall, episode of care unspecified | Cardiac | 3,881 | 4.8% |
| 429.9 | Heart disease, unspecified | Cardiac | 3,798 | 4.7% |
| 428.9 | Heart failure, unspecified | Cardiac | 3,715 | 4.6% |
| 416.8 | Other chronic pulmonary heart diseases | Cardiac | 3,492 | 4.3% |
| 428.23 | Acute on chronic systolic heart failure | Cardiac | 3,422 | 4.2% |
| 401.0 | Malignant essential hypertension | Cardiac | 3,370 | 4.2% |
| 729.81 | Swelling of limb | Cardiac | 3,226 | 4.0% |
| 272.4 | Other and unspecified hyperlipidemia | Cardiac - lipid | 31,712 | 39.2% |
| 272.0 | Pure hypercholesterolemia | Cardiac - lipid | 6,367 | 7.9% |
| 427,31 | Atrial fibrillation | Cardiac - rhythm | 14,660 | 18.1% |
| 427,89 | Other specified cardiac dysrhythmias | Cardiac - rhythm | 12,702 | 15.7% |
| 427.1 | Paroxysmal ventricular tachycardia | Cardiac - rhythm | 5,583 | 6.9% |
| 427.9 | Cardiac dysrhythmia, unspecified | Cardiac - rhythm | 4,908 | 6.1% |
| 427.5 | Cardiac arrest | Cardiac - rhythm | 3,633 | 4.5% |
| 424.0 | Mitral valve disorders | Cardiac - valve | 11,268 | 13.9% |
| 424.1 | Aortic valve disorders | Cardiac - valve | 6,472 | 8.0% |
| 424.2 | Tricuspid valve disorders, specified as nonrheumatic | Cardiac - valve | 4,774 | 5.9% |
| 250.00 | Diabetes mellitus without mention of complication, type II or unspecified type, not stated as uncontrolled | Endocrine | 20,440 | 25.3% |
| 780.79 | Other malaise and fatigue | Endocrine | 6,907 | 8.5% |
| 244.9 | Unspecified hypothyroidism | Endocrine | 5,980 | 7.4% |
| 250.02 | Diabetes mellitus without mention of complication, type II or unspecified type, uncontrolled | Endocrine | 4,935 | 6.1% |
| V58.82 | Fitting and adjustment of nonvascular catheter, NEC | Factors Influencing Health Status | 7,353 | 9.1% |
| V58.81 | Fitting and adjustment of vascular catheter | Factors Influencing Health Status | 6,827 | 8.4% |
| V45.82 | Percutaneous transluminal coronary angioplasty status | Factors Influencing Health Status | 6,375 | 7.9% |
| V45.81 | Aortocoronary bypass status | Factors Influencing Health Status | 6,174 | 7.6% |
| V15.82 | Personal history of tobacco use, presenting hazards to health | Factors Influencing Health Status | 4,309 | 5.3% |
| V45.89 | Other postprocedural status | Factors Influencing Health Status | 3,786 | 4.7% |
| V17.3 | Family history of ischemic heart disease | Factors Influencing Health Status | 3,374 | 4.2% |
| 530.81 | Esophageal reflux | Gastrointestinal | 7,393 | 9.1% |
| 789.00 | Abdominal pain, unspecified site | Gastrointestinal | 5,177 | 6.4% |
| 787.01 | Nausea with vomiting | Gastrointestinal | 3,410 | 4.2% |
| 285.9 | Anemia, unspecified | Hemic | 8,360 | 10.3% |
| 288.60 | Leukocytosis, unspecified | Hemic | 5,036 | 6.2% |
| 285.1 | Acute post hemorrhagic anemia | Hemic | 4,436 | 5.5% |
| 486 | Pneumonia, organism unspecified | Infectious disease | 9,241 | 11.4% |
| 038.9 | Unspecified septicemia | Infectious disease | 5,612 | 6.9% |
| 599.0 | Urinary tract infection, site not specified | Infectious disease | 5,011 | 6.2% |
| 780.60 | Fever, unspecified | Infectious disease | 4,117 | 5.1% |
| 995.91 | Sepsis | Infectious disease | 3,784 | 4.7% |
| 278.00 | Obesity, unspecified | Metabolic | 6,090 | 7.5% |
| 790.5 | Other nonspecific abnormal serum enzyme levels | Metabolic - lab | 8,111 | 10.0% |
| 790.6 | Other abnormal blood chemistry | Metabolic - lab | 5,963 | 7.4% |
| 276.8 | Hypopotassemia | Metabolic - lab | 5,330 | 6.6% |
| 276.2 | Acidosis | Metabolic - lab | 4,462 | 5.5% |
| 276.1 | Hypoosmolality and/or hyponatremia | Metabolic - lab | 4,432 | 5.5% |
| 790.99 | Other nonspecific findings on examination of blood | Metabolic - lab | 3,806 | 4.7% |
| 729.5 | Pain in limb | Musculoskeletal | 4,535 | 5.6% |
| 305.1 | Tobacco use disorder | Neuropsychiatric | 11,584 | 14.3% |
| 780.97 | Altered mental status | Neuropsychiatric | 5,868 | 7.3% |
| 300.00 | Anxiety state, unspecified | Neuropsychiatric | 3,898 | 4.8% |
| 780.4 | Dizziness and giddiness | Neuropsychiatric | 3,415 | 4.2% |
| 780.09 | Other alteration of consciousness | Neuropsychiatric | 3,288 | 4.1% |
| 786.05 | Shortness of breath | Pulmonary | 24,563 | 30.4% |
| 786.09 | Other dyspnea and respiratory abnormality | Pulmonary | 13,378 | 16.6% |
| 511.9 | Unspecified pleural effusion | Pulmonary | 12,276 | 15.2% |
| 518.81 | Acute respiratory failure | Pulmonary | 12,123 | 15.0% |
| 518.0 | Pulmonary collapse | Pulmonary | 11,537 | 14.3% |
| 793.19 | Other nonspecific abnormal finding of lung field | Pulmonary | 9,833 | 12.2% |
| 496 | Chronic airway obstruction, not elsewhere classified | Pulmonary | 8,373 | 10.4% |
| 514 | Pulmonary congestion and hypostasis | Pulmonary | 7,734 | 9.6% |
| 799.02 | Hypoxemia | Pulmonary | 5,596 | 6.9% |
| 518.89 | Other diseases of lung, not elsewhere classified | Pulmonary | 4,969 | 6.1% |
| 786.2 | Cough | Pulmonary | 4,401 | 5.4% |
| 327.23 | Obstructive sleep apnea (adult) (pediatric) | Pulmonary | 3,860 | 4.8% |
| 491.21 | Obstructive chronic bronchitis, with (acute) exacerbation | Pulmonary | 3,211 | 4.0% |
| 584.9 | Acute kidney failure, unspecified | Renal | 13,856 | 17.1% |
| 585.9 | Chronic kidney disease, unspecified | Renal | 4,399 | 5.4% |
| 403.90 | Hypertensive chronic kidney disease, unspecified, with chronic kidney disease stage I through stage IV, or unspecified | Renal | 4,331 | 5.4% |
| 585.3 | Chronic kidney disease, Stage III (moderate) | Renal | 4,231 | 5.2% |
| 433.10 | Occlusion and stenosis of carotid artery without mention of cerebral infarction | Vascular | 3,285 | 4.1% |
| 434.91 | Unspecified cerebral artery occlusion with cerebral infarction | Vascular | 3,281 | 4.1% |
| 443.9 | Peripheral vascular disease, unspecified | Vascular | 3,262 | 4.0% |
| **ICD-10-CM** | | | **N** | **% of Total (95,017)** |
| I21.4 | Non-ST elevation (NSTEMI) myocardial infarction | Cardiac | 65,789 | 69.2% |
| I10 | Essential (primary) hypertension | Cardiac | 59,538 | 62.7% |
| R07.9 | Chest pain, unspecified | Cardiac | 55,153 | 58.0% |
| I25.10 | Atherosclerotic heart disease native coronary artery w/o angina pectoris | Cardiac | 53,677 | 56.5% |
| I21.3 | ST elevation (STEMI) myocardial infarction of unspecified site | Cardiac | 27,211 | 28.6% |
| R07.89 | Other chest pain | Cardiac | 23,606 | 24.8% |
| R94.31 | Abnormal electrocardiogram [ECG] [EKG] | Cardiac | 23,012 | 24.2% |
| I50.9 | Heart failure, unspecified | Cardiac | 17,673 | 18.6% |
| I51.7 | Cardiomegaly | Cardiac | 14,921 | 15.7% |
| I25.110 | ASHD of native coronary artery with unstable angina pectoris | Cardiac | 12,790 | 13.5% |
| I21.19 | ST elevation myocardial infarction involving other coronary artery inferior wall | Cardiac | 11,761 | 12.4% |
| R00.0 | Tachycardia, unspecified | Cardiac | 9,382 | 9.9% |
| I21.09 | ST elevation myocardial infarction involving other coronary artery of ant wall | Cardiac | 9,241 | 9.7% |
| I20.0 | Unstable angina | Cardiac | 8,488 | 8.9% |
| I95.9 | Hypotension, unspecified | Cardiac | 8,335 | 8.8% |
| I25.5 | Ischemic cardiomyopathy | Cardiac | 7,529 | 7.9% |
| I24.9 | Acute ischemic heart disease, unspecified | Cardiac | 7,427 | 7.8% |
| R00.1 | Bradycardia, unspecified | Cardiac | 7,421 | 7.8% |
| R07.2 | Precordial pain | Cardiac | 6,941 | 7.3% |
| I50.21 | Acute systolic (congestive) heart failure | Cardiac | 6,852 | 7.2% |
| I42.9 | Cardiomyopathy, unspecified | Cardiac | 6,419 | 6.8% |
| I25.2 | Old myocardial infarction | Cardiac | 6,369 | 6.7% |
| I25.119 | ASHD of native coronary artery with unspecified angina pectoris | Cardiac | 6,349 | 6.7% |
| R55 | Syncope and collapse | Cardiac | 5,674 | 6.0% |
| I11.0 | Hypertensive heart disease with heart failure | Cardiac | 5,306 | 5.6% |
| I21.11 | ST elevation (STEMI) myocardial infarct involving right coronary artery | Cardiac | 5,210 | 5.5% |
| I21.02 | ST elevation myocardial infarct involving left ant descending coronary art | Cardiac | 4,994 | 5.3% |
| I50.23 | Acute on chronic systolic (congestive) heart failure | Cardiac | 4,719 | 5.0% |
| I25.82 | Chronic total occlusion of coronary artery | Cardiac | 4,655 | 4.9% |
| R57.0 | Cardiogenic shock | Cardiac | 4,244 | 4.5% |
| I21.29 | ST elevation (STEMI) myocardial infarction involving other sites | Cardiac | 4,218 | 4.4% |
| I27.2 | Other secondary pulmonary hypertension | Cardiac | 4,164 | 4.4% |
| I20.9 | Angina pectoris, unspecified | Cardiac | 4,153 | 4.4% |
| R09.89 | Other specified symptoms and signs involving circulatory and resp systems | Cardiac - and pulmonary | 5,621 | 5.9% |
| E78.5 | Hyperlipidemia, unspecified | Cardiac - lipid | 37,216 | 39.2% |
| E78.0 | Pure hypercholesterolemia | Cardiac - lipid | 6,036 | 6.4% |
| E78.2 | Mixed hyperlipidemia | Cardiac - lipid | 4,606 | 4.8% |
| I48.91 | Unspecified atrial fibrillation | Cardiac - rhythm | 13,618 | 14.3% |
| I48.0 | Paroxysmal atrial fibrillation | Cardiac - rhythm | 8,867 | 9.3% |
| I47.2 | Ventricular tachycardia | Cardiac - rhythm | 6,199 | 6.5% |
| I49.9 | Cardiac arrhythmia, unspecified | Cardiac - rhythm | 5,351 | 5.6% |
| I46.9 | Cardiac arrest, cause unspecified | Cardiac - rhythm | 4,638 | 4.9% |
| I34.0 | Nonrheumatic mitral (valve) insufficiency | Cardiac - valve | 10,661 | 11.2% |
| I36.1 | Nonrheumatic tricuspid (valve) insufficiency | Cardiac - valve | 5,309 | 5.6% |
| I35.0 | Nonrheumatic aortic (valve) stenosis | Cardiac - valve | 4,239 | 4.5% |
| E11.9 | Type 2 diabetes mellitus without complications | Endocrine | 21,440 | 22.6% |
| E11.65 | Type 2 diabetes mellitus with hyperglycemia | Endocrine | 7,998 | 8.4% |
| E03.9 | Hypothyroidism, unspecified | Endocrine | 7,534 | 7.9% |
| Z46.82 | Encounter for fitting and adjustment of non-vascular catheter | Factors influencing health status | 9,777 | 10.3% |
| Z45.2 | Encounter for adjustment and management of vascular access device | Factors influencing health status | 8,447 | 8.9% |
| Z95.1 | Presence of aortocoronary bypass graft | Factors influencing health status | 6,784 | 7.1% |
| Z87.891 | Personal history of nicotine dependence | Factors influencing health status | 6,654 | 7.0% |
| Z95.5 | Presence of coronary angioplasty implant and graft | Factors influencing health status | 5,225 | 5.5% |
| Z79.82 | Long term (current) use of aspirin | Factors influencing health status | 4,742 | 5.0% |
| Z82.49 | Family Hx of ischemic heart disease & other diseases of circulatory system | Factors influencing health status | 4,726 | 5.0% |
| Z98.89 | Other specified postprocedural state | Factors influencing health status | 4,660 | 4.9% |
| Z74.3 | Need for continuous supervision | Factors influencing health status | 4,572 | 4.8% |
| Z79.899 | Other long-term drug therapy | Factors influencing health status | 4,356 | 4.6% |
| K21.9 | Gastro-esophageal reflux disease without esophagitis | Gastrointestinal | 9,237 | 9.7% |
| R10.9 | Unspecified abdominal pain | Gastrointestinal | 5,539 | 5.8% |
| D64.9 | Anemia, unspecified | Hemic | 10,254 | 10.8% |
| D72.829 | Elevated white blood cell count, unspecified | Hemic | 7,018 | 7.4% |
| D62 | Acute post hemorrhagic anemia | Hemic | 5,934 | 6.2% |
| D69.6 | Thrombocytopenia, unspecified | Hemic | 4,434 | 4.7% |
| J18.9 | Pneumonia, unspecified organism | Infectious disease | 11,027 | 11.6% |
| A41.9 | Sepsis, unspecified organism | Infectious disease | 9,269 | 9.8% |
| N39.0 | Urinary tract infection, site not specified | Infectious disease | 5,726 | 6.0% |
| R50.9 | Fever, unspecified | Infectious disease | 4,752 | 5.0% |
| E66.9 | Obesity, unspecified | Metabolic | 7,854 | 8.3% |
| R53.1 | Weakness | Metabolic | 7,642 | 8.0% |
| E66.01 | Morbid (severe) obesity due to excess calories | Metabolic | 4,592 | 4.8% |
| R79.89 | Other specified abnormal finding of blood chemistry | Metabolic - lab | 13,297 | 14.0% |
| E87.2 | Acidosis | Metabolic - lab | 8,140 | 8.6% |
| R74.8 | Abnormal levels of other serum enzymes | Metabolic - lab | 8,112 | 8.5% |
| E87.6 | Hypokalemia | Metabolic - lab | 7,122 | 7.5% |
| E87.1 | Hypo-osmolality and hyponatremia | Metabolic - lab | 5,651 | 5.9% |
| F17.210 | Nicotine dependence, cigarettes, uncomplicated | Neuropsychiatric | 8,707 | 9.2% |
| R41.82 | Altered mental status, unspecified | Neuropsychiatric | 7,074 | 7.4% |
| F41.9 | Anxiety disorder, unspecified | Neuropsychiatric | 4,973 | 5.2% |
| G93.40 | Encephalopathy, unspecified | Neuropsychiatric | 4,078 | 4.3% |
| R06.02 | Shortness of breath | Pulmonary | 28,918 | 30.4% |
| R91.8 | Other nonspecific abnormal finding of lung field | Pulmonary | 15,813 | 16.6% |
| J90 | Pleural effusion, not elsewhere classified | Pulmonary | 15,429 | 16.2% |
| J98.11 | Atelectasis | Pulmonary | 14,305 | 15.1% |
| R06.00 | Dyspnea, unspecified | Pulmonary | 11,866 | 12.5% |
| J96.01 | Acute respiratory failure with hypoxia | Pulmonary | 11,529 | 12.1% |
| J44.9 | Chronic obstructive pulmonary disease, unspecified | Pulmonary | 9,193 | 9.7% |
| J96.00 | Acute respiratory failure, unspecified whether hypoxia or hypercapnia | Pulmonary | 7,922 | 8.3% |
| J81.1 | Chronic pulmonary edema | Pulmonary | 7,567 | 8.0% |
| R09.02 | Hypoxemia | Pulmonary | 6,638 | 7.0% |
| J96.90 | Respiratory failure, unspecified, unspecified whether hypoxia or hypercapnia | Pulmonary | 5,895 | 6.2% |
| R05 | Cough | Pulmonary | 5,548 | 5.8% |
| G47.33 | Obstructive sleep apnea (adult) (pediatric) | Pulmonary | 5,434 | 5.7% |
| J98.4 | Other disorders of lung | Pulmonary | 4,614 | 4.9% |
| J44.1 | Chronic obstructive pulmonary disease with (acute) exacerbation | Pulmonary | 4,205 | 4.4% |
| N17.9 | Acute kidney failure, unspecified | Renal | 18,251 | 19.2% |
| I12.9 | Hypertensive chronic kidney disease stage 1 through stage 4 or unspecified | Renal | 6,367 | 6.7% |
| N18.3 | Chronic kidney disease, stage 3 (moderate) | Renal | 5,880 | 6.2% |
| N18.9 | Chronic kidney disease, unspecified | Renal | 4,925 | 5.2% |
| E86.0 | Dehydration | Renal | 4,409 | 4.6% |

**Table C3** lists the 100 drug products most commonly prescribed one day before and 14 days following a first AMI diagnosis among a cohort of 268,424 patients receiving ≥1 AMI diagnosis code in any healthcare setting between 2014–2017. Treatments are organized alphabetically by class and by the most commonly reported treatment within each class.

**Table C3.** Most common drug products prescribed from one day before to 14 days following first AMI diagnosis (any healthcare setting).

| **Drug Product** | **Class** | **Category** | **Persons (N)** | **% of Total** |
| --- | --- | --- | --- | --- |
| Atorvastatin Calcium | Cardiac | HMG CoA reductase inhibitor - statin | 75,983 | 28.3% |
| Clopidogrel Hydrogen Sulfate | Cardiac | Antiplatelet | 47,489 | 17.7% |
| Metoprolol Tartrate | Cardiac | HTN - beta-blocker | 45,765 | 17.0% |
| Lisinopril | Cardiac | HTN - ACE/ARB | 43,995 | 16.4% |
| Nitroglycerin | Cardiac | Antianginal - nitrate | 40,237 | 15.0% |
| Carvedilol | Cardiac | HTN - alpha+beta-blocker | 29,493 | 11.0% |
| Metoprolol Succinate | Cardiac | HTN - beta-blocker | 27,871 | 10.4% |
| Furosemide | Cardiac | HTN - diuretic | 22,976 | 8.6% |
| Ticagrelor | Cardiac | Antiplatelet | 18,884 | 7.0% |
| Aspirin | Cardiac | Antiplatelet | 14,808 | 5.5% |
| Amlodipine Besylate | Cardiac | HTN - calcium channel blocker | 14,152 | 5.3% |
| Isosorbide Mononitrate | Cardiac | Antianginal - nitrate | 12,163 | 4.5% |
| Losartan Potassium | Cardiac | HTN - ACE/ARB | 10,092 | 3.8% |
| Prasugrel Hydrochloride | Cardiac | Antiplatelet | 9,482 | 3.5% |
| Simvastatin | Cardiac | HMG CoA reductase inhibitor - statin | 7,918 | 2.9% |
| Rosuvastatin Calcium | Cardiac | HMG CoA reductase inhibitor - statin | 7,619 | 2.8% |
| Amiodarone Hydrochloride | Cardiac | Antiarrhythmic | 7,572 | 2.8% |
| Spironolactone | Cardiac | HTN - diuretic | 6,914 | 2.6% |
| Pravastatin Sodium | Cardiac | HMG CoA reductase inhibitor - statin | 6,477 | 2.4% |
| Diltiazem Hydrochloride | Cardiac | HTN - calcium channel blocker | 4,138 | 1.5% |
| Ramipril | Cardiac | HTN - ACE/ARB | 3,926 | 1.5% |
| Hydrochlorothiazide | Cardiac | HTN - diuretic | 3,894 | 1.5% |
| Hydralazine Hydrochloride | Cardiac | HTN - diuretic | 3,823 | 1.4% |
| Atenolol | Cardiac | HTN - beta-blocker | 3,035 | 1.1% |
| Digoxin | Cardiac | Cardiac glycoside | 2,692 | 1.0% |
| Ranolazine | Cardiac | Antianginal - metabolic modulator | 2,544 | 0.9% |
| Valsartan | Cardiac | HTN - ACE/ARB | 2,450 | 0.9% |
| Enalapril Maleate | Cardiac | HTN - ACE/ARB | 2,193 | 0.8% |
| Fenofibrate | Cardiac | Fibrate | 2,067 | 0.8% |
| Ezetimibe | Cardiac | Cholesterol lowering agent | 1,855 | 0.7% |
| Nifedipine | Cardiac | HTN - calcium channel blocker | 1,713 | 0.6% |
| Clonidine Hydrochloride | Cardiac | HTN - centrally acting alpha-agonist | 1,677 | 0.6% |
| Bumetanide | Cardiac | HTN - diuretic | 1,428 | 0.5% |
| Hydrochlorothiazide/Lisinopril | Cardiac | HTN - ACE/ARB diuretic combo | 1,396 | 0.5% |
| Metformin Hydrochloride | Endocrine | DM - oral | 9,592 | 3.6% |
| Levothyroxine Sodium | Endocrine | Thyroid Hormone | 8,728 | 3.3% |
| Prednisone | Endocrine | Corticosteroid | 8,202 | 3.1% |
| Insulin Glargine, Recombinant | Endocrine | DM - insulin | 4,955 | 1.8% |
| Glucose Meter Test Control Strips | Endocrine | DM - device | 4,579 | 1.7% |
| Insulin Syringe/Needle | Endocrine | DM - device | 2,957 | 1.1% |
| Lancet | Endocrine | DM - device | 2,600 | 1.0% |
| Glipizide | Endocrine | DM - oral | 2,452 | 0.9% |
| Insulin Aspart, Recombinant | Endocrine | DM - insulin | 2,252 | 0.8% |
| Glimepiride | Endocrine | DM - oral | 2,009 | 0.7% |
| Methylprednisolone | Endocrine | Corticosteroid | 1,942 | 0.7% |
| Insulin Detemir | Endocrine | DM - insulin | 1,814 | 0.7% |
| Insulin Lispro, Recombinant | Endocrine | DM - insulin | 1,773 | 0.7% |
| Sitagliptin Phosphate | Endocrine | DM - DPP-4 inhibitor | 1,709 | 0.6% |
| Needle | Endocrine | DM - device | 1,619 | 0.6% |
| Pantoprazole Sodium | Gastrointestinal | Proton pump inhibitor | 14,487 | 5.4% |
| Omeprazole | Gastrointestinal | Proton pump inhibitor | 7,243 | 2.7% |
| Famotidine | Gastrointestinal | H2 - blocker | 3,484 | 1.3% |
| Ondansetron Hydrochloride | Gastrointestinal | 5-HT3 antagonist | 1,816 | 0.7% |
| Ranitidine Hydrochloride | Gastrointestinal | H2 - blocker | 1,763 | 0.7% |
| Ondansetron | Gastrointestinal | 5-HT3 antagonist | 1,483 | 0.6% |
| Tamsulosin Hydrochloride | Genitourinary | Prostate - Alpha-1 blocker | 4,536 | 1.7% |
| Warfarin Sodium | Hemic | Anticoagulant | 7,843 | 2.9% |
| Apixaban | Hemic | Anticoagulant | 3,551 | 1.3% |
| Rivaroxaban | Hemic | Anticoagulant | 3,352 | 1.2% |
| Enoxaparin Sodium | Hemic | Anticoagulant | 2,565 | 1.0% |
| Levofloxacin | Infectious disease | Antibiotic | 6,251 | 2.3% |
| Ciprofloxacin Hydrochloride | Infectious disease | Antibiotic | 3,829 | 1.4% |
| Azithromycin | Infectious disease | Antibiotic | 3,686 | 1.4% |
| Cephalexin | Infectious disease | Antibiotic | 3,431 | 1.3% |
| Amoxicillin/Clavulanate Potassium | Infectious disease | Antibiotic | 3,382 | 1.3% |
| Doxycycline Hyclate | Infectious disease | Antibiotic | 2,154 | 0.8% |
| Sulfamethoxazole/ Trimethoprim | Infectious disease | Antibiotic | 2,115 | 0.8% |
| Amoxicillin | Infectious disease | Antibiotic | 1,925 | 0.7% |
| Metronidazole | Infectious disease | Antibiotic | 1,551 | 0.6% |
| Allopurinol | Metabolic | Anti-gout - xanthine oxidase inhibitor | 2,492 | 0.9% |
| Colchicine | Metabolic | Anti-gout - microtubule polymerization inhibitor | 1,941 | 0.7% |
| Alprazolam | Neuropsychiatric | Benzodiazepine | 5,900 | 2.2% |
| Zolpidem Tartrate | Neuropsychiatric | Benzodiazepine-like | 3,073 | 1.1% |
| Lorazepam | Neuropsychiatric | Benzodiazepine | 3,043 | 1.1% |
| Sertraline Hydrochloride | Neuropsychiatric | Antidepressant - SSRI | 2,754 | 1.0% |
| Escitalopram Oxalate | Neuropsychiatric | Antidepressant - SSRI | 2,173 | 0.8% |
| Citalopram Hydrobromide | Neuropsychiatric | Antidepressant - SSRI | 2,093 | 0.8% |
| Bupropion Hydrochloride | Neuropsychiatric | Antidepressant - SSRI | 1,945 | 0.7% |
| Trazodone Hydrochloride | Neuropsychiatric | Antidepressant - SARI | 1,870 | 0.7% |
| Duloxetine Hydrochloride | Neuropsychiatric | Antidepressant - SNRI | 1,666 | 0.6% |
| Clonazepam | Neuropsychiatric | Benzodiazepine | 1,654 | 0.6% |
| Nicotine | Neuropsychiatric | Smoking cessation aid | 1,541 | 0.6% |
| Potassium Chloride | Nutritional | Electrolyte - potassium | 12,933 | 4.8% |
| Ergocalciferol | Nutritional | Vitamin D | 1,906 | 0.7% |
| Folic Acid | Nutritional | Vitamin B | 1,625 | 0.6% |
| Albuterol Sulfate | Pulmonary | Inhaled - beta-agonist | 6,049 | 2.3% |
| Fluticasone Propionate | Pulmonary | Inhaled - corticosteroid | 2,581 | 1.0% |
| Tiotropium Bromide | Pulmonary | Inhaled - anticholinergic | 1,901 | 0.7% |
| Montelukast Sodium | Pulmonary | Leukotriene receptor antagonist | 1,798 | 0.7% |
| Albuterol Sulfate/Ipratropium Bromide | Pulmonary | Inhaled - beta-agonist/anticholinergic | 1,696 | 0.6% |
| Fluticasone Propionate/Salmeterol Xinafoate | Pulmonary | Inhaled - corticosteroid/beta-agonist | 1,640 | 0.6% |
| Benzonatate | Pulmonary | Antitussive | 1,527 | 0.6% |
| Acetaminophen/Hydrocodone Bitartrate | Symptomatic | Analgesic | 14,690 | 5.5% |
| Acetaminophen/Oxycodone Hydrochloride | Symptomatic | Analgesic | 7,858 | 2.9% |
| Tramadol Hydrochloride | Symptomatic | Analgesic | 5,350 | 2.0% |
| Gabapentin | Symptomatic | Analgesic - anticonvulsant | 4,857 | 1.8% |
| Oxycodone Hydrochloride | Symptomatic | Analgesic | 4,412 | 1.6% |
| Cyclobenzaprine Hydrochloride | Symptomatic | Muscle relaxant - non-benzodiazepine | 1,724 | 0.6% |
| Ibuprofen | Symptomatic | Analgesic | 1,678 | 0.6% |
| Acetaminophen/Codeine Phosphate | Symptomatic | Analgesic | 1,575 | 0.6% |
|  |  |  |  |  |

**Table C4** lists the 100 drug products most commonly prescribed one day before and 14 days following a first AMI diagnosis among a cohort of 170,147 patients receiving an inpatient AMI diagnosis. Treatments are organized alphabetically by class then by the most commonly reported treatment within each class.

**Table C4.** Most common drug products prescribed from one day before to 14 days following first AMI diagnosis (inpatient setting).

| **Drug Product** | **Class** | **Category** | **Persons** | **% of Total** |
| --- | --- | --- | --- | --- |
| Atorvastatin Calcium | Cardiac | HMG CoA reductase inhibitor - statin | 62,281 | 36.6% |
| Clopidogrel Hydrogen Sulfate | Cardiac | Antiplatelet | 38,750 | 22.8% |
| Metoprolol Tartrate | Cardiac | HTN - beta-blocker | 38,190 | 22.4% |
| Lisinopril | Cardiac | HTN - ACE/ARB | 35,398 | 20.8% |
| Nitroglycerin | Cardiac | Antianginal - nitrate | 34,351 | 20.2% |
| Carvedilol | Cardiac | HTN - alpha+beta-blocker | 23,359 | 13.7% |
| Metoprolol Succinate | Cardiac | HTN - beta-blocker | 21,012 | 12.3% |
| Furosemide | Cardiac | HTN - diuretic | 17,713 | 10.4% |
| Ticagrelor | Cardiac | Antiplatelet | 17,029 | 10.0% |
| Aspirin | Cardiac | Antiplatelet | 12,931 | 7.6% |
| Amlodipine Besylate | Cardiac | HTN - calcium channel blocker | 9,547 | 5.6% |
| Isosorbide Mononitrate | Cardiac | Antianginal - nitrate | 9,182 | 5.4% |
| Prasugrel Hydrochloride | Cardiac | Antiplatelet | 8,052 | 4.7% |
| Losartan Potassium | Cardiac | HTN - ACE/ARB | 7,091 | 4.2% |
| Amiodarone Hydrochloride | Cardiac | Antiarrhythmic | 6,370 | 3.7% |
| Spironolactone | Cardiac | HTN - diuretic | 5,244 | 3.1% |
| Rosuvastatin Calcium | Cardiac | HMG CoA reductase inhibitor - statin | 5,104 | 3.0% |
| Simvastatin | Cardiac | HMG CoA reductase inhibitor - statin | 4,957 | 2.9% |
| Pravastatin Sodium | Cardiac | HMG CoA reductase inhibitor - statin | 4,615 | 2.7% |
| Hydralazine Hydrochloride | Cardiac | HTN - diuretic | 2,865 | 1.7% |
| Ramipril | Cardiac | HTN - ACE/ARB | 2,850 | 1.7% |
| Diltiazem Hydrochloride | Cardiac | HTN - calcium channel blocker | 2,763 | 1.6% |
| Hydrochlorothiazide | Cardiac | HTN - diuretic | 2,366 | 1.4% |
| Atenolol | Cardiac | HTN - beta-blocker | 1,845 | 1.1% |
| Digoxin | Cardiac | Cardiac glycoside | 1,821 | 1.1% |
| Ranolazine | Cardiac | Antianginal - metabolic modulator | 1,772 | 1.0% |
| Valsartan | Cardiac | HTN - ACE/ARB | 1,688 | 1.0% |
| Enalapril Maleate | Cardiac | HTN - ACE/ARB | 1,636 | 1.0% |
| Fenofibrate | Cardiac | Fibrate | 1,325 | 0.8% |
| Nifedipine | Cardiac | HTN - calcium channel blocker | 1,176 | 0.7% |
| Clonidine Hydrochloride | Cardiac | HTN - centrally acting alpha-agonist | 1,127 | 0.7% |
| Bumetanide | Cardiac | HTN – diuretic | 1,072 | 0.6% |
| Ezetimibe | Cardiac | Cholesterol lowering agent | 1,027 | 0.6% |
| Metformin Hydrochloride | Endocrine | DM - oral | 6,469 | 3.8% |
| Prednisone | Endocrine | Corticosteroid | 5,732 | 3.4% |
| Levothyroxine Sodium | Endocrine | Thyroid Hormone | 5,233 | 3.1% |
| Insulin Glargine, Recombinant | Endocrine | DM - insulin | 3,586 | 2.1% |
| Glucose Meter Test Control Strips | Endocrine | DM - device | 3,426 | 2.0% |
| Insulin Syringe/Needle | Endocrine | DM - device | 2,194 | 1.3% |
| Lancet | Endocrine | DM - device | 2,112 | 1.2% |
| Glipizide | Endocrine | DM - oral | 1,679 | 1.0% |
| Insulin Aspart, Recombinant | Endocrine | DM - insulin | 1,664 | 1.0% |
| Insulin Detemir | Endocrine | DM - insulin | 1,394 | 0.8% |
| Glimepiride | Endocrine | DM - oral | 1,343 | 0.8% |
| Insulin Lispro, Recombinant | Endocrine | DM - insulin | 1,324 | 0.8% |
| Methylprednisolone | Endocrine | Corticosteroid | 1,225 | 0.7% |
| Needle | Endocrine | DM - device | 1,188 | 0.7% |
| Sitagliptin Phosphate | Endocrine | DM - DPP-4 inhibitor | 1,123 | 0.7% |
| Pantoprazole Sodium | Gastrointestinal | Proton pump inhibitor | 10,854 | 6.4% |
| Omeprazole | Gastrointestinal | Proton pump inhibitor | 4,457 | 2.6% |
| Famotidine | Gastrointestinal | H2 - blocker | 2,775 | 1.6% |
| Ondansetron Hydrochloride | Gastrointestinal | 5-HT3 antagonist | 1,125 | 0.7% |
| Ranitidine Hydrochloride | Gastrointestinal | H2 - blocker | 1,125 | 0.7% |
| Ondansetron | Gastrointestinal | 5-HT3 antagonist | 923 | 0.5% |
| Polyethylene Glycol 3350 | Gastrointestinal | Laxative | 910 | 0.5% |
| Tamsulosin Hydrochloride | Genitourinary | Prostate - Alpha-1 blocker | 2,869 | 1.7% |
| Warfarin Sodium | Hemic | Anticoagulant | 5,311 | 3.1% |
| Apixaban | Hemic | Anticoagulant | 2,538 | 1.5% |
| Rivaroxaban | Hemic | Anticoagulant | 2,291 | 1.3% |
| Enoxaparin Sodium | Hemic | Anticoagulant | 1,898 | 1.1% |
| Levofloxacin | Infectious disease | Antibiotic | 4,716 | 2.8% |
| Ciprofloxacin Hydrochloride | Infectious disease | Antibiotic | 2,406 | 1.4% |
| Amoxicillin/Clavulanate Potassium | Infectious disease | Antibiotic | 2,403 | 1.4% |
| Azithromycin | Infectious disease | Antibiotic | 2,319 | 1.4% |
| Cephalexin | Infectious disease | Antibiotic | 2,240 | 1.3% |
| Doxycycline Hyclate | Infectious disease | Antibiotic | 1,464 | 0.9% |
| Sulfamethoxazole/Trimethoprim | Infectious disease | Antibiotic | 1,320 | 0.8% |
| Amoxicillin | Infectious disease | Antibiotic | 1,043 | 0.6% |
| Metronidazole | Infectious disease | Antibiotic | 1,028 | 0.6% |
| Cefuroxime Axetil | Infectious disease | Antibiotic | 982 | 0.6% |
| Allopurinol | Metabolic | Anti-gout - xanthine oxidase inhibitor | 1,455 | 0.9% |
| Colchicine | Metabolic | Anti-gout - microtubule polymerization inhibitor | 1,411 | 0.8% |
| Alprazolam | Neuropsychiatric | Benzodiazepine | 3,960 | 2.3% |
| Lorazepam | Neuropsychiatric | Benzodiazepine | 1,967 | 1.2% |
| Zolpidem Tartrate | Neuropsychiatric | Benzodiazepine-like | 1,835 | 1.1% |
| Sertraline Hydrochloride | Neuropsychiatric | Antidepressant - SSRI | 1,654 | 1.0% |
| Nicotine | Neuropsychiatric | Smoking cessation aid | 1,387 | 0.8% |
| Escitalopram Oxalate | Neuropsychiatric | Antidepressant - SSRI | 1,282 | 0.8% |
| Citalopram Hydrobromide | Neuropsychiatric | Antidepressant - SSRI | 1,248 | 0.7% |
| Bupropion Hydrochloride | Neuropsychiatric | Antidepressant - SSRI | 1,223 | 0.7% |
| Trazodone Hydrochloride | Neuropsychiatric | Antidepressant - SARI | 1,148 | 0.7% |
| Clonazepam | Neuropsychiatric | Benzodiazepine | 981 | 0.6% |
| Duloxetine Hydrochloride | Neuropsychiatric | Antidepressant - SNRI | 935 | 0.5% |
| Potassium Chloride | Nutritional | Electrolyte | 10,067 | 5.9% |
| Ergocalciferol | Nutritional | Vitamin D | 1,141 | 0.7% |
| Folic Acid | Nutritional | Vitamin B | 1,109 | 0.7% |
| Albuterol Sulfate | Pulmonary | Inhaled - beta-agonist | 4,078 | 2.4% |
| Fluticasone Propionate | Pulmonary | Inhaled - corticosteroid | 1,450 | 0.9% |
| Tiotropium Bromide | Pulmonary | Inhaled - anticholinergic | 1,322 | 0.8% |
| Albuterol Sulfate/Ipratropium Bromide | Pulmonary | Inhaled - beta-agonist/anticholinergic | 1,238 | 0.7% |
| Fluticasone Propionate/Salmeterol Xinafoate | Pulmonary | Inhaled - corticosteroid/beta-agonist | 1,073 | 0.6% |
| Benzonatate | Pulmonary | Antitussive | 1,064 | 0.6% |
| Montelukast Sodium | Pulmonary | Leukotriene receptor antagonist | 999 | 0.6% |
| Acetaminophen/Hydrocodone Bitartrate | Symptomatic | Analgesic | 9,775 | 5.7% |
| Acetaminophen/Oxycodone Hydrochloride | Symptomatic | Analgesic | 5,441 | 3.2% |
| Tramadol Hydrochloride | Symptomatic | Analgesic | 3,622 | 2.1% |
| Oxycodone Hydrochloride | Symptomatic | Analgesic | 3,134 | 1.8% |
| Gabapentin | Symptomatic | Analgesic - anticonvulsant | 2,847 | 1.7% |
| Acetaminophen/Codeine Phosphate | Symptomatic | Analgesic | 1,002 | 0.6% |
| Cyclobenzaprine Hydrochloride | Symptomatic | Muscle relaxant - non-benzodiezipine | 901 | 0.5% |

**Table C5** lists the procedures most commonly reported one day before and 14 days following a first AMI diagnosis among a cohort of 268,424 patients receiving ≥1 AMI diagnosis code in any healthcare setting between 2014–2017. Procedures are organized alphabetically by category then by the most commonly reported codes within each category.

**Table C5.** Most common procedures codes received one day before to 14 days following first AMI diagnosis (any healthcare setting).

| **Code** | **CPT/HCPCS – Procedure Description** | **Category** | **Persons (N)** | **% of Total** |
| --- | --- | --- | --- | --- |
| 99232 | Subsequent hospital care, per day, for the evaluation and management of a patient, which requires at least 2 of these 3 key components: An expanded problem focused interval history; An expanded problem focused examination | E/M | 143,466 | 53.4% |
| 99223 | Initial hospital care, per day, for the evaluation and management of a patient, which requires these 3 key components: A comprehensive history; A comprehensive examination; and Medical decision making of high complexity. | E/M | 139,725 | 52.0% |
| 99285 | Emergency department visit for the evaluation and management of a patient, which requires these 3 key components within the constraints imposed by the urgency of the patient's clinical condition and/or mental status: A comprehensive history | E/M | 124,184 | 46.2% |
| 99233 | Subsequent hospital care, per day, for the evaluation and management of a patient, which requires at least 2 of these 3 key components: A detailed interval history; A detailed examination; Medical decision making of high complexity. | E/M | 113,648 | 42.3% |
| 99291 | Critical care, evaluation and management of the critically ill or critically injured patient; first 30-74 minutes | E/M | 91,543 | 34.1% |
| 99214 | Office or other outpatient visit for the evaluation and management of an established patient, which requires at least 2 of these 3 key components: A detailed history; A detailed examination; Medical decision making of moderate complexity. | E/M | 90,752 | 33.8% |
| 99239 | Hospital discharge day management; more than 30 minutes | E/M | 77,816 | 29.0% |
| 99222 | Initial hospital care, per day, for the evaluation and management of a patient, which requires these 3 key components: A comprehensive history; A comprehensive examination; and Medical decision making of moderate complexity. | E/M | 74,374 | 27.7% |
| 99238 | Hospital discharge day management; 30 minutes or less | E/M | 66,132 | 24.6% |
| 99213 | Office or other outpatient visit for the evaluation and management of an established patient, which requires at least 2 of these 3 key components: An expanded problem focused history; An expanded problem focused examination | E/M | 50,580 | 18.8% |
| 99231 | Subsequent hospital care, per day, for the evaluation and management of a patient, which requires at least 2 of these 3 key components: A problem focused interval history; A problem focused examination; Medical decision making that is straightforward | E/M | 46,970 | 17.5% |
| 99221 | Initial hospital care, per day, for the evaluation and management of a patient, which requires these 3 key components: A detailed or comprehensive history; A detailed or comprehensive examination | E/M | 23,240 | 8.6% |
| 99254 | Inpatient consultation for a new or established patient, which requires these 3 key components: A comprehensive history; A comprehensive examination; and Medical decision making of moderate complexity. | E/M | 22,612 | 8.4% |
| 99284 | Emergency department visit for the evaluation and management of a patient, which requires these 3 key components: A detailed history; A detailed examination; and Medical decision making of moderate complexity. | E/M | 19,940 | 7.4% |
| 99255 | Inpatient consultation for a new or established patient, which requires these 3 key components: A comprehensive history; A comprehensive examination; and Medical decision making of high complexity. | E/M | 19,691 | 7.3% |
| 99215 | Office or other outpatient visit for the evaluation and management of an established patient, which requires at least 2 of these 3 key components: A comprehensive history; A comprehensive examination; Medical decision making of high complexity. | E/M | 17,481 | 6.5% |
| 99220 | Initial observation care, per day, for the evaluation and management of a patient, which requires these 3 key components: A comprehensive history; A comprehensive examination; and Medical decision making of high complexity. | E/M | 15,140 | 5.6% |
| 99292 | Critical care, evaluation and management of the critically ill or critically injured patient; each additional 30 minutes (List separately in addition to code for primary service) | E/M | 12,128 | 4.5% |
| 99204 | Office or other outpatient visit for the evaluation and management of a new patient, which requires these 3 key components: A comprehensive history; A comprehensive examination; Medical decision making of moderate complexity. | E/M | 11,201 | 4.2% |
| 99253 | Inpatient consultation for a new or established patient, which requires these 3 key components: A detailed history; A detailed examination; and Medical decision making of low complexity. Counseling and/or coordination of care with other physicians | E/M | 10,038 | 3.7% |
| 99152 | Moderate sedation services provided by the same physician or other qualified health care professional performing the diagnostic or therapeutic service that the sedation supports, requiring the presence of an independent trained observer to assist | E/M - anesthesia | 9,933 | 3.7% |
| G0378 | Hospital observation service, per hour | E/M - HCPCS code | 12,799 | 4.8% |
| G8427 | Eligible clinician attests to documenting in the medical record they obtained, updated, or reviewed the patient's current medications | E/M - HCPCS code | 10,013 | 3.7% |
| A0425 | Ground mileage, per statute mile | EMS | 108,741 | 40.5% |
| A0427 | Ambulance service, advanced life support, emergency transport, level 1 (als 1 - emergency) | EMS | 74,359 | 27.7% |
| A0428 | Ambulance service, basic life support, non-emergency transport, (bls) | EMS | 14,562 | 5.4% |
| A0426 | Ambulance service, advanced life support, non-emergency transport, level 1 (als 1) | EMS | 14,523 | 5.4% |
| A0429 | Ambulance service, basic life support, emergency transport (bls-emergency) | EMS | 12,790 | 4.8% |
| 70450 | Computed tomography, head or brain; without contrast material | Imaging - advanced | 37,208 | 13.8% |
| 71275 | Computed tomographic angiography, chest (noncoronary), with contrast material(s), including noncontrast images, if performed, and image postprocessing | Imaging - advanced | 21,876 | 8.1% |
| 74176 | Computed tomography, abdomen and pelvis; without contrast material | Imaging - advanced | 12,829 | 4.8% |
| 71250 | Computed tomography, thorax; without contrast material | Imaging - advanced | 12,079 | 4.5% |
| 74177 | Computed tomography, abdomen and pelvis; with contrast material(s) | Imaging - advanced | 10,250 | 3.8% |
| 71260 | Computed tomography, thorax; with contrast material(s) | Imaging - advanced | 10,238 | 3.8% |
| 93306 | Echocardiography, transthoracic, real-time with image documentation (2D), includes M-mode recording, when performed, complete, with spectral Doppler echocardiography, and with color flow Doppler echocardiography | Imaging - cardiac | 145,009 | 54.0% |
| 93458 | Catheter placement in coronary artery(s) for coronary angiography, including intraprocedural injection(s) for coronary angiography, imaging supervision and interpretation; with left heart catheterization including intraprocedural injection(s) | Imaging - cardiac | 92,541 | 34.4% |
| 88.53 | Other diagnostic radiology and related techniques: Angiocardiography of left heart structures; Angiocardiography of: aortic valve left atrium left ventricle (outflow tract) | Imaging - cardiac | 21,641 | 8.1% |
| 78452 | Myocardial perfusion imaging, tomographic (SPECT) (including attenuation correction, qualitative or quantitative wall motion, ejection fraction by first pass or gated technique, additional quantification, when performed) | Imaging - cardiac | 19,857 | 7.4% |
| 88.56 | Other diagnostic radiology and related techniques: Coronary arteriography using two catheters; Coronary arteriography by: Judkins technique; Ricketts and Abrams technique; Direct selective coronary arteriography using two catheters | Imaging - cardiac | 19,406 | 7.2% |
| 93325 | Doppler echocardiography color flow velocity mapping (List separately in addition to codes for echocardiography) | Imaging - cardiac | 13,595 | 5.1% |
| 93312 | Echocardiography, transesophageal, real-time with image documentation (2D) (with or without M-mode recording); including probe placement, image acquisition, interpretation and report | Imaging - cardiac | 12,527 | 4.7% |
| 93308 | Echocardiography, transthoracic, real-time with image documentation (2D), includes M-mode recording, when performed, follow-up or limited study | Imaging - cardiac | 11,432 | 4.3% |
| 93010 | Electrocardiogram, routine ECG with at least 12 leads; interpretation and report only | Imaging - cardiac ECG report | 191,167 | 71.1% |
| 93005 | Electrocardiogram, routine ECG with at least 12 leads; tracing only, without interpretation and report | Imaging - cardiac ECG tracing | 53,638 | 20.0% |
| 93000 | Electrocardiogram, routine ECG with at least 12 leads; with interpretation and report | Imaging - cardiac ECG tracing + report | 44,095 | 16.4% |
| 93018 | Cardiovascular stress test using maximal or submaximal treadmill or bicycle exercise, continuous electrocardiographic monitoring, and/or pharmacological stress; interpretation and report only | Imaging - cardiac testing | 18,057 | 6.7% |
| 93016 | Cardiovascular stress test using maximal or submaximal treadmill or bicycle exercise, continuous electrocardiographic monitoring, and/or pharmacological stress; supervision only, without interpretation and report | Imaging - cardiac testing | 15,270 | 5.7% |
| 71010 | Radiologic examination, chest; single view, frontal | Imaging - plain | 156,756 | 58.3% |
| 71020 | Radiologic examination, chest, 2 views, frontal and lateral; | Imaging - plain | 70,736 | 26.3% |
| 74000 | Radiologic examination, abdomen; single anteroposterior view | Imaging - plain | 14,319 | 5.3% |
| 85025 | Blood count; complete (CBC), automated (Hgb, Hct, RBC, WBC and platelet count) and automated differential WBC count | Laboratory | 66,038 | 24.6% |
| 36415 | Collection of venous blood by venipuncture | Laboratory | 57,722 | 21.5% |
| 84484 | Troponin, quantitative | Laboratory | 56,802 | 21.1% |
| 80053 | Comprehensive metabolic panel This panel must include the following: Albumin (82040) Bilirubin, total (82247) Calcium, total (82310) Carbon dioxide (bicarbonate) (82374) Chloride (82435) Creatinine (82565) Glucose (82947) Phosphatase, alkaline (84075 | Laboratory | 55,141 | 20.5% |
| 85610 | Prothrombin time; | Laboratory | 47,799 | 17.8% |
| 80048 | Basic metabolic panel (Calcium, total) This panel must include the following: Calcium, total (82310) Carbon dioxide (bicarbonate) (82374) Chloride (82435) Creatinine (82565) Glucose (82947) Potassium (84132) Sodium (84295) Urea nitrogen (BUN) (84520) | Laboratory | 45,463 | 16.9% |
| 85730 | Thromboplastin time, partial (PTT); plasma or whole blood | Laboratory | 35,901 | 13.4% |
| 82550 | Creatine kinase (CK), (CPK); total | Laboratory | 29,630 | 11.0% |
| 83735 | Magnesium | Laboratory | 28,680 | 10.7% |
| 85027 | Blood count; complete (CBC), automated (Hgb, Hct, RBC, WBC and platelet count) | Laboratory | 25,619 | 9.5% |
| 83036 | Hemoglobin; glycosylated (A1C) | Laboratory | 19,592 | 7.3% |
| 84443 | Thyroid stimulating hormone (TSH) | Laboratory | 16,069 | 6.0% |
| 81001 | Urinalysis, by dip stick or tablet reagent for bilirubin, glucose, hemoglobin, ketones, leukocytes, nitrite, pH, protein, specific gravity, urobilinogen, any number of these constituents; automated, with microscopy | Laboratory | 15,215 | 5.7% |
| 83690 | Lipase | Laboratory | 10,352 | 3.9% |
| 85379 | Fibrin degradation products, D-dimer; quantitative | Laboratory | 9,521 | 3.5% |
| 80061 | Lipid panel This panel must include the following: Cholesterol, serum, total (82465) Lipoprotein, direct measurement, high density cholesterol (HDL cholesterol) (83718) Triglycerides (84478) | Laboratory - cardiac | 33,326 | 12.4% |
| 82553 | Creatine kinase (CK), (CPK); MB fraction only | Laboratory - cardiac | 25,711 | 9.6% |
| 83880 | Natriuretic peptide | Laboratory - cardiac | 23,395 | 8.7% |
| 96374 | Therapeutic, prophylactic, or diagnostic injection (specify substance or drug); intravenous push, single or initial substance/drug | Medication/med management | 19,475 | 7.2% |
| 96375 | Therapeutic, prophylactic, or diagnostic injection (specify substance or drug); each additional sequential intravenous push of a new substance/drug (List separately in addition to code for primary procedure) | Medication/med management | 16,532 | 6.2% |
| Q9967 | Low osmolar contrast material, 300-399 mg/ml iodine concentration, per ml | Medication/med management | 14,048 | 5.2% |
| 96365 | Intravenous infusion, for therapy, prophylaxis, or diagnosis (specify substance or drug); initial, up to 1 hour | Medication/med management | 10,493 | 3.9% |
| 96372 | Therapeutic, prophylactic, or diagnostic injection (specify substance or drug); subcutaneous or intramuscular | Medication/med management | 10,176 | 3.8% |
| 88305 | Level IV - Surgical pathology, gross and microscopic examination Abortion - spontaneous/missed Artery, biopsy Bone marrow, biopsy Bone exostosis Brain/meninges, other than for tumor resection Breast, biopsy, not requiring microscopic evaluation | Pathology | 13,639 | 5.1% |
| 36620 | Arterial catheterization or cannulation for sampling, monitoring or transfusion (separate procedure); percutaneous | Procedure | 20,852 | 7.8% |
| 36556 | Insertion of non-tunneled centrally inserted central venous catheter; age 5 years or older | Procedure | 16,946 | 6.3% |
| 31500 | Intubation, endotracheal, emergency procedure | Procedure | 10,941 | 4.1% |
| 92928 | Percutaneous transcatheter placement of intracoronary stent(s), with coronary angioplasty when performed; single major coronary artery or branch | Procedure - cardiac | 38,810 | 14.4% |
| 37.22 | Other operations on heart and pericardium: Left heart cardiac catheterization | Procedure - cardiac | 34,744 | 12.9% |
| 92941 | Percutaneous transluminal revascularization of acute total/subtotal occlusion during acute myocardial infarction, coronary artery or coronary artery bypass graft, any combination of intracoronary stent, atherectomy and angioplasty | Procedure - cardiac | 33,870 | 12.6% |
| 00.66 | Procedures and interventions, Not Elsewhere Classified: Percutaneous transluminal coronary angioplasty [PTCA]; Balloon angioplasty of coronary artery; Percutaneous coronary angioplasty NOS; PTCA NOS | Procedure - cardiac | 31,751 | 11.8% |
| 36.07 | Operations on vessels of heart: Insertion of drug-eluting coronary artery stent(s); Endograft(s); Endovascular graft(s); Stent graft(s) | Procedure - cardiac | 21,872 | 8.1% |
| 93454 | Catheter placement in coronary artery(s) for coronary angiography, including intraprocedural injection(s) for coronary angiography, imaging supervision and interpretation; | Procedure - cardiac | 15,844 | 5.9% |
| 00.40 | Procedures and interventions, Not Elsewhere Classified: Procedure on single vessel; Number of vessels, unspecified | Procedure - cardiac | 13,846 | 5.2% |
| 33533 | Coronary artery bypass, using arterial graft(s); single arterial graft | Procedure - cardiac | 12,051 | 4.5% |
| 93503 | Insertion and placement of flow directed catheter (e.g., Swan-Ganz) for monitoring purposes | Procedure - cardiac | 11,799 | 4.4% |
| 00.45 | Procedures and interventions, Not Elsewhere Classified: Insertion of one vascular stent; Number of stents, unspecified | Procedure - cardiac | 11,593 | 4.3% |
| 00567 | Anesthesia for direct coronary artery bypass grafting; with pump oxygenator | Procedure - cardiac | 11,111 | 4.1% |
| 33508 | Endoscopy, surgical, including video-assisted harvest of vein(s) for coronary artery bypass procedure (List separately in addition to code for primary procedure) | Procedure - cardiac | 10,661 | 4.0% |
| 93880 | Duplex scan of extracranial arteries; complete bilateral study | Ultrasound/doppler | 19,489 | 7.3% |
| 93970 | Duplex scan of extremity veins including responses to compression and other maneuvers; complete bilateral study | Ultrasound/doppler | 19,044 | 7.1% |
| 76937 | Ultrasound guidance for vascular access requiring ultrasound evaluation of potential access sites, documentation of selected vessel patency, concurrent real-time ultrasound visualization of vascular needle entry, with permanent recording and reporting | Ultrasound/doppler | 12,376 | 4.6% |
| 76770 | Ultrasound, retroperitoneal (e.g., renal, aorta, nodes), real time with image documentation; complete | Ultrasound/doppler | 9,621 | 3.6% |

Acronyms: CPT, Current Procedural Terminology; E/M, evaluation and management; EMS, emergency medical services; HCPCS, Healthcare Common Procedure Coding System

**Table C6** lists the procedures most commonly prescribed one day before and 14 days following a first AMI diagnosis among a cohort of 170,147 patients receiving an AMI diagnosis in an inpatient setting. Procedures are organized alphabetically by category then by the most commonly reported codes within each category.

**Table C6.** Most common procedure codes from one day before to 14 days following first AMI diagnosis (inpatient setting).

| **Code** | **CPT/HCPCS – Procedure Description** | **Category** | **Persons (N)** | **% of Total** |
| --- | --- | --- | --- | --- |
| 99232 | Subsequent hospital care, per day, for the evaluation and management of a patient, which requires at least 2 of these 3 key components: An expanded problem focused interval history; An expanded problem focused examination | E/M | 124,961 | 73.3% |
| 99223 | Initial hospital care, per day, for the evaluation and management of a patient, which requires these 3 key components: A comprehensive history; A comprehensive examination; and Medical decision making of high complexity. | E/M | 122,181 | 71.7% |
| 99233 | Subsequent hospital care, per day, for the evaluation and management of a patient, which requires at least 2 of these 3 key components: A detailed interval history; A detailed examination; Medical decision making of high complexity. | E/M | 99,353 | 58.3% |
| 99285 | Emergency department visit for the evaluation and management of a patient, which requires these 3 key components within the constraints imposed by the urgency of the patient's clinical condition and/or mental status: A comprehensive history | E/M | 95,821 | 56.2% |
| 99291 | Critical care, evaluation and management of the critically ill or critically injured patient; first 30-74 minutes | E/M | 79,313 | 46.5% |
| 99239 | Hospital discharge day management; more than 30 minutes | E/M | 68,286 | 40.1% |
| 99222 | Initial hospital care, per day, for the evaluation and management of a patient, which requires these 3 key components: A comprehensive history; A comprehensive examination; and Medical decision making of moderate complexity. | E/M | 62,984 | 37.0% |
| 99238 | Hospital discharge day management; 30 minutes or less | E/M | 58,536 | 34.3% |
| 99214 | Office or other outpatient visit for the evaluation and management of an established patient, which requires at least 2 of these 3 key components: A detailed history; A detailed examination; Medical decision making of moderate complexity. Counseling | E/M | 51,881 | 30.4% |
| 99231 | Subsequent hospital care, per day, for the evaluation and management of a patient, which requires at least 2 of these 3 key components: A problem focused interval history; A problem focused examination; Medical decision making that is straightforward | E/M | 40,446 | 23.7% |
| 99213 | Office or other outpatient visit for the evaluation and management of an established patient, which requires at least 2 of these 3 key components: An expanded problem focused history; An expanded problem focused examination | E/M | 27,186 | 16.0% |
| 99254 | Inpatient consultation for a new or established patient, which requires these 3 key components: A comprehensive history; A comprehensive examination; and Medical decision making of moderate complexity. | E/M | 21,001 | 12.3% |
| 99221 | Initial hospital care, per day, for the evaluation and management of a patient, which requires these 3 key components: A detailed or comprehensive history; A detailed or comprehensive examination | E/M | 19,815 | 11.6% |
| 99255 | Inpatient consultation for a new or established patient, which requires these 3 key components: A comprehensive history; A comprehensive examination; and Medical decision making of high complexity. | E/M | 18,551 | 10.9% |
| 99284 | Emergency department visit for the evaluation and management of a patient, which requires these 3 key components: A detailed history; A detailed examination; and Medical decision making of moderate complexity. | E/M | 12,477 | 7.3% |
| 99292 | Critical care, evaluation and management of the critically ill or critically injured patient; each additional 30 minutes (List separately in addition to code for primary service) | E/M | 10,756 | 6.3% |
| 99215 | Office or other outpatient visit for the evaluation and management of an established patient, which requires at least 2 of these 3 key components: A comprehensive history; A comprehensive examination; Medical decision making of high complexity. | E/M | 10,553 | 6.2% |
| 99253 | Inpatient consultation for a new or established patient, which requires these 3 key components: A detailed history; A detailed examination; and Medical decision making of low complexity. | E/M | 9,357 | 5.5% |
| 99152 | Moderate sedation services provided by the same physician or other qualified health care professional performing the diagnostic or therapeutic service that the sedation supports, requiring the presence of an independent trained observer to assist | E/M | 8,368 | 4.9% |
| 99053 | Service(s) provided between 10:00 PM and 8:00 AM at 24-hour facility, in addition to basic service | E/M | 7,426 | 4.4% |
| 99220 | Initial observation care, per day, for the evaluation and management of a patient, which requires these 3 key components: A comprehensive history; A comprehensive examination; and Medical decision making of high complexity. | E/M | 7,109 | 4.2% |
| 00567 | Anesthesia for direct coronary artery bypass grafting; with pump oxygenator | E/M - anesthesia | 10,556 | 6.2% |
| G8427 | Eligible clinician attests to documenting in the medical record they obtained, updated, or reviewed the patient's current medications | E/M - HCPCS | 7,017 | 4.1% |
| A0425 | Ground mileage, per statute mile | EMS | 87,856 | 51.5% |
| A0427 | Ambulance service, advanced life support, emergency transport, level 1 (als 1 - emergency) | EMS | 60,479 | 35.5% |
| A0426 | Ambulance service, advanced life support, non-emergency transport, level 1 (als 1) | EMS | 12,278 | 7.2% |
| A0428 | Ambulance service, basic life support, non-emergency transport, (bls) | EMS | 10,240 | 6.0% |
| A0429 | Ambulance service, basic life support, emergency transport (bls-emergency) | EMS | 9,645 | 5.7% |
| A0434 | Specialty care transport (sct) | EMS | 6,363 | 3.7% |
| A0422 | Ambulance (als or bls) oxygen and oxygen supplies, life sustaining situation | EMS | 6,290 | 3.7% |
| 70450 | Computed tomography, head or brain; without contrast material | Imaging - advanced | 29,155 | 17.1% |
| 71275 | Computed tomographic angiography, chest (noncoronary), with contrast material(s), including noncontrast images, if performed, and image postprocessing | Imaging - advanced | 17,518 | 10.3% |
| 74176 | Computed tomography, abdomen and pelvis; without contrast material | Imaging - advanced | 10,433 | 6.1% |
| 71250 | Computed tomography, thorax; without contrast material | Imaging - advanced | 9,973 | 5.9% |
| 71260 | Computed tomography, thorax; with contrast material(s) | Imaging - advanced | 8,111 | 4.8% |
| 74177 | Computed tomography, abdomen and pelvis; with contrast material(s) | Imaging - advanced | 8,105 | 4.8% |
| 93306 | Echocardiography, transthoracic, real-time with image documentation (2D), includes M-mode recording, when performed, complete, with spectral Doppler echocardiography, and with color flow Doppler echocardiography | Imaging - cardiac | 119,881 | 70.3% |
| 93458 | Catheter placement in coronary artery(s) for coronary angiography, including intraprocedural injection(s) for coronary angiography, imaging supervision and interpretation; with left heart catheterization including intraprocedural injection(s) | Imaging - cardiac | 80,577 | 47.3% |
| 88.53 | Other diagnostic radiology and related techniques: Angiocardiography of left heart structures; Angiocardiography of: aortic valve left atrium left ventricle (outflow tract) | Imaging - cardiac | 21,296 | 12.5% |
| 88.56 | Other diagnostic radiology and related techniques: Coronary arteriography using two catheters; Coronary arteriography by: Judkins technique; Ricketts and Abrams technique; Direct selective coronary arteriography using two catheters | Imaging - cardiac | 19,000 | 11.1% |
| 93454 | Catheter placement in coronary artery(s) for coronary angiography, including intraprocedural injection(s) for coronary angiography, imaging supervision and interpretation; | Imaging - cardiac | 13,892 | 8.2% |
| 93325 | Doppler echocardiography color flow velocity mapping (List separately in addition to codes for echocardiography) | Imaging - cardiac | 11,087 | 6.5% |
| 93503 | Insertion and placement of flow directed catheter (e.g., Swan-Ganz) for monitoring purposes | Imaging - cardiac | 11,070 | 6.5% |
| 93312 | Echocardiography, transesophageal, real-time with image documentation (2D) (with or without M-mode recording); including probe placement, image acquisition, interpretation and report | Imaging - cardiac | 10,999 | 6.5% |
| 78452 | Myocardial perfusion imaging, tomographic (SPECT) (including attenuation correction, qualitative or quantitative wall motion, ejection fraction by first pass or gated technique, additional quantification, when performed) | Imaging - cardiac | 10,141 | 5.9% |
| 93308 | Echocardiography, transthoracic, real-time with image documentation (2D), includes M-mode recording, when performed, follow-up or limited study | Imaging - cardiac | 9,782 | 5.7% |
| 93459 | Catheter placement in coronary artery(s) for coronary angiography, including intraprocedural injection(s) for coronary angiography, imaging supervision and interpretation; with left heart catheterization including intraprocedural injection(s) | Imaging - cardiac | 7,624 | 4.5% |
| 93320 | Doppler echocardiography, pulsed wave and/or continuous wave with spectral display (List separately in addition to codes for echocardiographic imaging); complete | Imaging - cardiac | 7,189 | 4.2% |
| 93010 | Electrocardiogram, routine ECG with at least 12 leads; interpretation and report only | Imaging - cardiac ECG report | 146,810 | 86.1% |
| 93005 | Electrocardiogram, routine ECG with at least 12 leads; tracing only, without interpretation and report | Imaging - cardiac ECG tracing | 28,343 | 16.6% |
| 93000 | Electrocardiogram, routine ECG with at least 12 leads; with interpretation and report | Imaging - cardiac ECG tracing and report | 26,082 | 15.3% |
| 93018 | Cardiovascular stress test using maximal or submaximal treadmill or bicycle exercise, continuous electrocardiographic monitoring, and/or pharmacological stress; interpretation and report only | Imaging - cardiac testing | 10,103 | 5.9% |
| 93016 | Cardiovascular stress test using maximal or submaximal treadmill or bicycle exercise, continuous electrocardiographic monitoring, and/or pharmacological stress; supervision only, without interpretation and report | Imaging - cardiac testing | 8,579 | 5.0% |
| 71010 | Radiologic examination, chest; single view, frontal | Imaging - plain | 128,602 | 75.5% |
| 71020 | Radiologic examination, chest, 2 views, frontal and lateral; | Imaging - plain | 54,520 | 32.0% |
| 74000 | Radiologic examination, abdomen; single anteroposterior view | Imaging - plain | 12,211 | 7.2% |
| 85025 | Blood count; complete (CBC), automated (Hgb, Hct, RBC, WBC and platelet count) and automated differential WBC count | Laboratory | 41,435 | 24.3% |
| 80053 | Comprehensive metabolic panel This panel must include the following: Albumin (82040) Bilirubin, total (82247) Calcium, total (82310) Carbon dioxide (bicarbonate) (82374) Chloride (82435) Creatinine (82565) Glucose (82947) Phosphatase, alkaline (84075 | Laboratory | 34,819 | 20.4% |
| 85610 | Prothrombin time; | Laboratory | 31,342 | 18.4% |
| 80048 | Basic metabolic panel (Calcium, total) This panel must include the following: Calcium, total (82310) Carbon dioxide (bicarbonate) (82374) Chloride (82435) Creatinine (82565) Glucose (82947) Potassium (84132) Sodium (84295) Urea nitrogen (BUN) (84520) | Laboratory | 28,597 | 16.8% |
| 85730 | Thromboplastin time, partial (PTT); plasma or whole blood | Laboratory | 24,781 | 14.5% |
| 82550 | Creatine kinase (CK), (CPK); total | Laboratory | 20,351 | 11.9% |
| 83735 | Magnesium | Laboratory | 19,646 | 11.5% |
| 80061 | Lipid panel This panel must include the following: Cholesterol, serum, total (82465) Lipoprotein, direct measurement, high density cholesterol (HDL cholesterol) (83718) Triglycerides (84478) | Laboratory | 17,596 | 10.3% |
| 85027 | Blood count; complete (CBC), automated (Hgb, Hct, RBC, WBC and platelet count) | Laboratory | 15,983 | 9.4% |
| 83036 | Hemoglobin; glycosylated (A1C) | Laboratory | 11,485 | 6.7% |
| 81001 | Urinalysis, by dip stick or tablet reagent for bilirubin, glucose, hemoglobin, ketones, leukocytes, nitrite, pH, protein, specific gravity, urobilinogen, any number of these constituents; automated, with microscopy | Laboratory | 9,430 | 5.5% |
| 84443 | Thyroid stimulating hormone (TSH) | Laboratory | 8,708 | 5.1% |
| 84100 | Phosphorus inorganic (phosphate); | Laboratory | 6,674 | 3.9% |
| 83690 | Lipase | Laboratory | 6,581 | 3.9% |
| 84484 | Troponin, quantitative | Laboratory - cardiac | 38,763 | 22.7% |
| 82553 | Creatine kinase (CK), (CPK); MB fraction only | Laboratory - cardiac | 18,254 | 10.7% |
| 83880 | Natriuretic peptide | Laboratory - cardiac | 16,106 | 9.4% |
| 96374 | Therapeutic, prophylactic, or diagnostic injection (specify substance or drug); intravenous push, single or initial substance/drug | Medication/med management | 12,181 | 7.1% |
| 96375 | Therapeutic, prophylactic, or diagnostic injection (specify substance or drug); each additional sequential intravenous push of a new substance/drug (List separately in addition to code for primary procedure) | Medication/med management | 11,479 | 6.7% |
| 96365 | Intravenous infusion, for therapy, prophylaxis, or diagnosis (specify substance or drug); initial, up to 1 hour | Medication/med management | 7,496 | 4.4% |
| J2270 | Injection, morphine sulfate, up to 10 mg | Medication/med management | 6,407 | 3.8% |
| 88305 | Level IV - Surgical pathology, gross and microscopic examination Abortion - spontaneous/missed Artery, biopsy Bone marrow, biopsy Bone exostosis Brain/meninges, other than for tumor resection Breast, biopsy, not requiring microscopic evaluation | Pathology | 9,568 | 5.6% |
| 36415 | Collection of venous blood by venipuncture | Procedure | 30,790 | 18.1% |
| 36620 | Arterial catheterization or cannulation for sampling, monitoring or transfusion (separate procedure); percutaneous | Procedure | 18,895 | 11.1% |
| 36556 | Insertion of non-tunneled centrally inserted central venous catheter; age 5 years or older | Procedure | 15,227 | 8.9% |
| 31500 | Intubation, endotracheal, emergency procedure | Procedure | 9,479 | 5.6% |
| 37.22 | Other operations on heart and pericardium: Left heart cardiac catheterization | Procedure - cardiac | 34,149 | 20.0% |
| 92928 | Percutaneous transcatheter placement of intracoronary stent(s), with coronary angioplasty when performed; single major coronary artery or branch | Procedure - cardiac | 33,883 | 19.9% |
| 92941 | Percutaneous transluminal revascularization of acute total/subtotal occlusion during acute myocardial infarction, coronary artery or coronary artery bypass graft, any combination of intracoronary stent, atherectomy and angioplasty | Procedure - cardiac | 31,880 | 18.7% |
| 00.66 | Procedures and interventions, Not Elsewhere Classified: Percutaneous transluminal coronary angioplasty [PTCA]; Balloon angioplasty of coronary artery; Percutaneous coronary angioplasty NOS; PTCA NOS | Procedure - cardiac | 31,494 | 18.5% |
| 36.07 | Operations on vessels of heart: Insertion of drug-eluting coronary artery stent(s); Endograft(s); Endovascular graft(s); Stent graft(s) | Procedure - cardiac | 21,651 | 12.7% |
| 00.40 | Procedures and interventions, Not Elsewhere Classified: Procedure on single vessel; Number of vessels, unspecified | Procedure - cardiac | 13,703 | 8.0% |
| 00.45 | Procedures and interventions, Not Elsewhere Classified: Insertion of one vascular stent; Number of stents, unspecified | Procedure - cardiac | 11,492 | 6.7% |
| 33533 | Coronary artery bypass, using arterial graft(s); single arterial graft | Procedure - cardiac | 11,402 | 6.7% |
| 33508 | Endoscopy, surgical, including video-assisted harvest of vein(s) for coronary artery bypass procedure (List separately in addition to code for primary procedure) | Procedure - cardiac | 10,128 | 5.9% |
| 92978 | Endoluminal imaging of coronary vessel or graft using intravascular ultrasound (IVUS) or optical coherence tomography (OCT) during diagnostic evaluation and/or therapeutic intervention including imaging supervision, interpretation and report; initial | Procedure - cardiac | 6,285 | 3.7% |
| 93880 | Duplex scan of extracranial arteries; complete bilateral study | Ultrasound/doppler | 16,106 | 9.4% |
| 93970 | Duplex scan of extremity veins including responses to compression and other maneuvers; complete bilateral study | Ultrasound/doppler | 15,953 | 9.4% |
| 76937 | Ultrasound guidance for vascular access requiring ultrasound evaluation of potential access sites, documentation of selected vessel patency, concurrent real-time ultrasound visualization of vascular needle entry, with permanent recording and reporting | Ultrasound/doppler | 10,969 | 6.4% |
| 76770 | Ultrasound, retroperitoneal (e.g., renal, aorta, nodes), real time with image documentation; complete | Ultrasound/doppler | 8,150 | 4.8% |
| 93971 | Duplex scan of extremity veins including responses to compression and other maneuvers; unilateral or limited study | Ultrasound/doppler | 7,058 | 4.1% |
| 76705 | Ultrasound, abdominal, real time with image documentation; limited (e.g., single organ, quadrant, follow-up) | Ultrasound/doppler | 7,019 | 4.1% |

Acronyms: CPT, Current Procedural Terminology; E/M, evaluation and management; EMS, emergency medical services; HCPCS, Healthcare Common Procedure Coding System
